# Rediscovering Cyanogen Gas for Organic Synthesis: Formation of 2-Cyanothiazole Derivatives

**DOI:** 10.1021/acs.joc.3c01110

**Published:** 2023-06-20

**Authors:** Michael Prieschl, Joerg Sedelmeier, Kurt Püntener, Stefan Hildbrand, Jason D. Williams, C. Oliver Kappe

**Affiliations:** † Center for Continuous Flow Synthesis and Processing (CC FLOW), Research Center Pharmaceutical Engineering GmbH (RCPE), Inffeldgasse 13, 8010 Graz, Austria; ‡ Institute of Chemistry, University of Graz, NAWI Graz, Heinrichstrasse 28, 8010 Graz, Austria; § Department of Process Chemistry & Catalysis, F. Hoffmann-La Roche Ltd, 4070 Basel, Switzerland

## Abstract

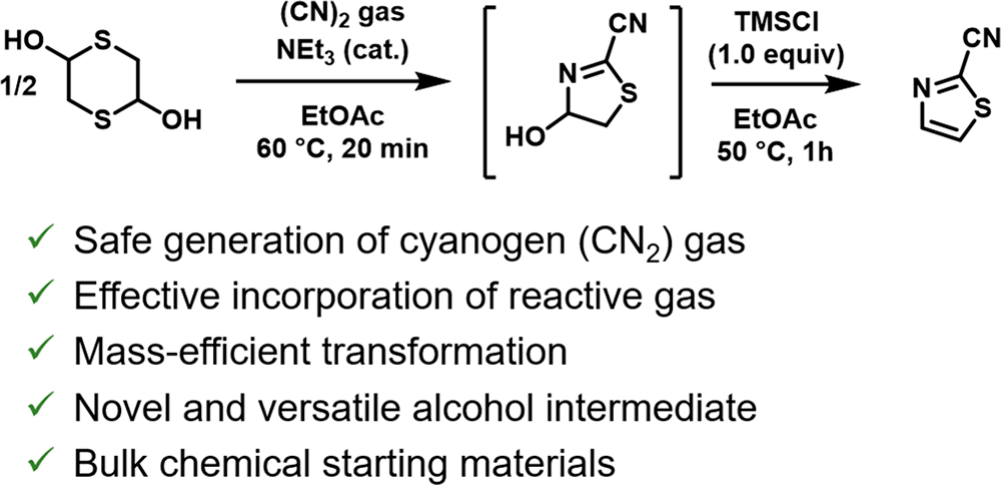

The expeditious synthesis of an API building block, 2-cyanothiazole, from cyanogen gas and a readily available dithiane is reported. A previously undisclosed partially saturated intermediate is formed, which can be further functionalized and isolated by the acylation of the hydroxy group. Dehydration using trimethylsilyl chloride furnished 2-cyanothiazole, which could be further converted to the corresponding amidine. The sequence provided a 55% yield over 4 steps. We envision that this work will spark further interest in cyanogen gas as a reactive and cost-effective synthetic reagent.

Thiazoles are important five-membered heterocycles, which are widely present in active pharmaceutical ingredients (APIs).^1^ The thiazole scaffold, when included in API structures, is often part of a fused bicyclic core and contains multiple points of functionalization. The direct and cost-effective synthesis of functionalized thiazoles is generally achieved via cyclization with a thioamide or thiourea (Scheme 1).^2^ This approach, however, generally requires additional alkyl/aryl ring substituents and limits the installation of a reactive functional group at the 2-position.

**Scheme 1:**
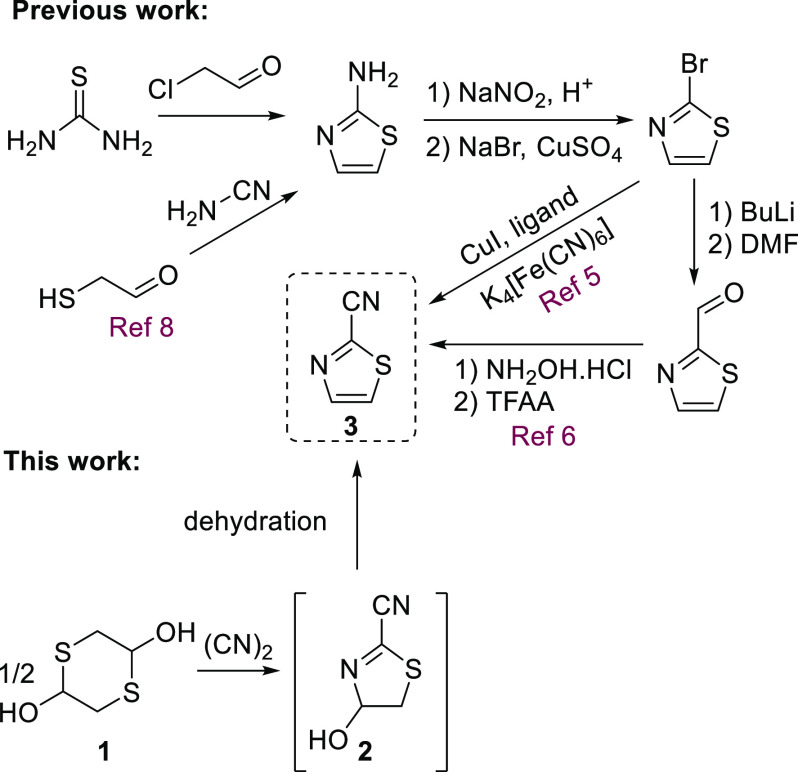
Synthesis of 2-Cyanothiazole **3** Previous work: numerous steps required to reach **3** from readily available materials. This work: straightforward access to **3** in two steps from readily available materials.

Of particular interest to us was the relatively simple 2-cyanothiazole, **3**, whose synthesis cannot be achieved through such a cyclization (Scheme 1). This nitrile-containing heterocycle is generally produced via the bromo precursor in a step-inefficient manner. The 2-aminothiazole is synthesized from readily available starting materials^3^ and then converted to 2-bromothiazole via a Sandmeyer reaction.^4^ Substitution of bromine by a nitrile group is then achieved by a copper-catalyzed coupling with ferricyanide,^5^ or via the aldehyde through lithium–halogen exchange.^6^

A recent publication demonstrated a variation of the Gewald reaction, in which 1,4-dithiane-2,5-diol **1** is reacted with nitrile-containing compounds to furnish 2-substituted thiazoles.^7^ Dithiane **1** is a dimeric form of mercaptoacetaldehyde, which possesses ambiphilic properties, making it an interesting component for sulfur-containing heterocycle formation. Notably, mercaptoacetaldehyde has also been demonstrated to react with cyanamide to form 2-aminothiazole in an analogous manner to the modified Gewald reaction (Scheme 1).^8^ We envisaged the reaction of **1** with cyanogen gas (ethanedinitrile, dicyan, (CN)_2_), to rapidly access thiazole **3**. Such an approach would utilize only readily available and cheap starting materials: dithiane **1**, a bulk chemical, which is frequently used to perform similar cyclizations,^9^ and (CN)_2_.

(CN)_2_ is a colorless and toxic gas, which was discovered by Gay-Lussac in 1816.^10^ Various cyclization reactions with (CN)_2_ have been reported, but recent literature is scarce.^11^ More recently, the use of (CN)_2_ as a fumigant for grains has been proposed, benefiting from its ready availability and rapid decomposition pathways.^12^ Laboratory scale preparations of (CN)_2_ are usually performed by thermal decomposition of metal cyanides or from oxidation of sodium cyanide (NaCN) with copper sulfate (CuSO_4_).^13^ On large scale (CN)_2_ is usually synthesized by the oxidation of hydrocyanic acid and is commercially available in large quantities.^14^

Herein, we report the synthesis of 2-cyanothiazole **3** from 1,4-dithiane-2,5-diol **1** and (CN)_2_. This proceeds via a previously unreported and versatile intermediate, **2** (4-hydroxy-4,5-dihydrothiazole-2-carbonitrile, Scheme 1). Furthermore, we showcase the functionalization of novel intermediate **2**, as well as the onward reaction of **3**.

Experiments were initiated by evaluating the optimal conditions and setup for the in situ generation of (CN)_2_ gas on a lab scale. Attempts to set up a “chemical generator” flow system,^15^ analogous to our previously reported setup for HCN,^16^ were unsuccessful, thought to be due to the high solubility of (CN)_2_. Additionally, we observed reactor clogging when forming (CN)_2_ in flow because of precipitation of CuCN, which is formed as a byproduct. As an alternative, we envisioned a simple ex situ generation setup using three batch vessels (Scheme 2, photograph in Supporting Information, Figure S1). In this setup (CN)_2_ was generated in the first flask, from CuSO_4_ and NaCN. The gas was then transferred under argon pressure from a balloon via perfluoroalkoxy (PFA) tubing (0.8 mm i.d.) and needles to a second flask, which contained the reaction solution.^17^ Any unreacted (CN)_2_ gas was then quenched in a third reaction vessel containing an aqueous NaOCl quench solution (adjusted to a pH of ∼10 with NaOH and NaHCO_3_).^18^ In contrast to published work, (CN)_2_ gas was directly used without any purification steps, which significantly simplified the setup.^13^

**Scheme 2:**
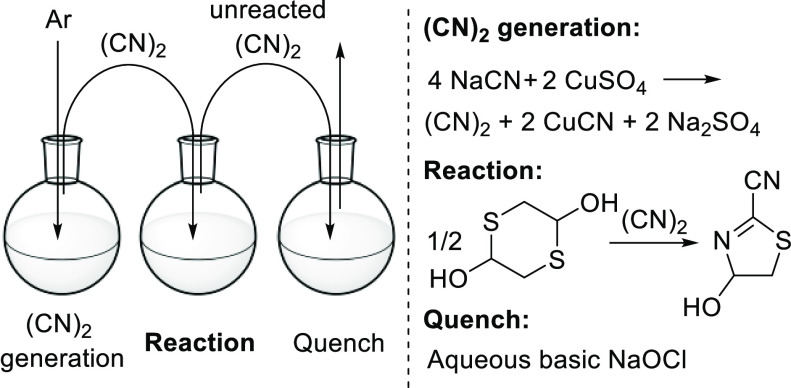
Reaction Setup for (CN)_2_ Addition to Dithiane **1** To Yield Intermediate **2**

To evaluate the efficiency of the (CN)_2_ formation, the previously reported reaction of (CN)_2_ with cysteamine was used as a model (Supporting Information, Table S1).^19^ Initially, (CN)_2_ was formed by the slow addition of an aqueous solution of CuSO_4_ (1 M) to a stirred and heated aqueous solution of NaCN (2 M). Doubling the concentration of NaCN (4 M) and CuSO_4_ (2 M) and reversing the addition order (NaCN added dropwise to CuSO_4_) provided the highest conversion of cysteamine (>80%). The improved performance at high concentration was most likely due to counteracting the high solubility of (CN)_2_.^10a^ We observed that the addition order was critical because of the potential reactivity of (CN)_2_ with excess cyanide ions to form polymers.

With a suitable protocol for (CN)_2_ generation in hand, our attention shifted to the reaction of dithiane **1** with (CN)_2_ in the hope of directly forming thiazole **3**. First attempts were conducted with EtOH as the reaction solvent, where the partially saturated intermediate **2** was observed as the major product (Table 1). Reaction of **1** with (CN)_2_ in the presence of *N*,*N*-diisopropylethylamine (DIPEA) led to 28% of **2** (entry 1). Since the formation of (CN)_2_ from NaCN and CuSO_4_ is reported to be only ∼80% efficient, a 2-fold excess of (CN)_2_ was employed, which led to an increase in product formation (41%, entry 2). GC analysis showed one major side product besides the desired product **2**. ^1^H NMR analysis of a crude reaction solution suggested that the side product was likely addition of EtOH to (CN)_2_ (Supporting Information, Figure S2).

**Table 1:**
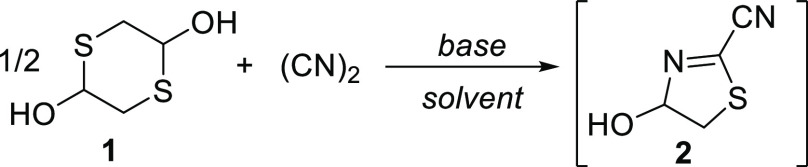
Optimization of Intermediate **2** Formation

Entry^a^	Solvent	*T* (°C)	Time (min)^b^	Base	**2** (%)^c^
1^d^	EtOH	30	15	DIPEA	28
2	EtOH	30	15	DIPEA	41
3	MeCN	30	30	DIPEA	12
4	PhMe	30	30	DIPEA	15
5	MeTHF	30	30	DIPEA	55
6	EtOAc	30	30	DIPEA	95
7	EtOAc	60	0	DIPEA	97
8	EtOAc	60	5	NEt_3_	92
9^e^	EtOAc	60	5	NEt_3_	95
10	EtOAc	60	15	–	37

a Standard reaction conditions: 1.0 equiv base; 2.0 equiv (CN)_2_ (assuming 100% yield of generation), 0.1 M concentration of **1**.

b Reaction time after dosing (CN)_2_ was finished.

c HPLC or GC calibrated yield against biphenyl as internal standard.

d 1.0 equiv of (CN)_2_.

e 0.1 equiv of base.

Accordingly, a screen was performed to identify a more suitable solvent (entries 3–6). Acetonitrile (MeCN) and toluene (PhMe) led to a low assay yield of **2** (12% and 15%, respectively). In 2-methyl-tetrahydrofuran (MeTHF) a higher yield of 55% was achieved. Reaction in ethyl acetate (EtOAc) led to an almost quantitative yield (95%) after 30 min. Increasing the reaction temperature to 60 °C resulted in a 97% HPLC yield directly after (CN)_2_ dosing was finished (entry 7).

It was also demonstrated that DIPEA could be replaced with the cheaper and more water-soluble tertiary amine triethylamine (NEt_3_), without a significant negative effect on the reaction outcome (entry 8). Finally, we were able to show that catalytic amounts of NEt_3_ (0.1 equiv) were sufficient to facilitate the reaction (entry 9). A control reaction without a base (entry 10) showed only low conversion to **2** (37%). This is expected since a catalytic base is reported to play a role in the monomerization of **1** to 2-mercaptoacetaldehyde.^20^ Experiments using a syringe pump to control (CN)_2_ generation showed that fast addition of (CN)_2_ was favorable (Supporting Information, Table S3). An addition time (NaCN to CuSO_4_) of 5 min was found to be suitable, while even faster addition times were not detrimental.

When we attempted to isolate compound **2** by evaporation of solvent, we observed degradation of the target compound as the concentration increased. By ^1^H NMR and GC-FID analysis, we were able to observe several new compounds (including desired product **3** as well as a pseudodimer) forming after evaporation of EtOAc (Supporting Information, Figure S3). Accordingly, we attempted to telescope the synthesis to the desired product **3** directly.

Product **2** was unstable not only in our isolation attempts but also when heated in EtOH solution (Supporting Information, Table S4). In contrast, **2** proved to be surprisingly stable in EtOAc, showing only traces of dehydration product **3** and 90% of **2** remaining after being heated to 100 °C for 2 h (Table 2, entry 1). Addition of a weak acid (AcOH) made no improvement in the formation of **3** (entry 2). Heating with HCl or H_2_SO_4_ led to almost full conversion of **2**, but poor selectivity was observed for **3** (entries 3 and 4). Methanesulfonyl chloride (MsCl) was added in an attempt to activate the alcohol toward elimination, which led to slightly improved product formation (50%, entry 5). Finally, trimethylsilyl chloride (TMSCl) provided high conversion and good selectivity for product **3** after just 15 min at 100 °C (72%, entry 6). Decreasing the temperature slightly improved the selectivity (76% entry 7). Finally, it was possible to reduce the TMSCl loading to 1 equiv without a loss of yield (75%, entry 8).

**Table 2:**
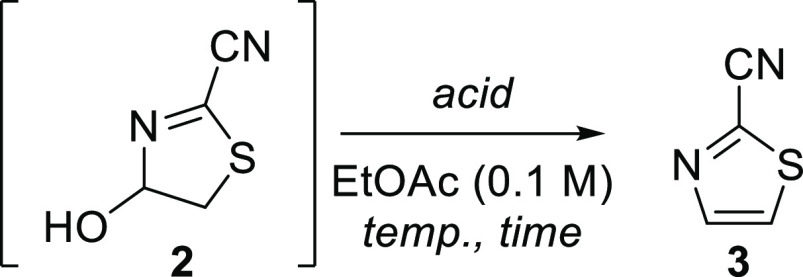
Optimization of Dehydration Reaction

Entry^a^	*T* (°C)	Time (min)	Acid (equiv)	**2**^b^ (%)	**3**^b^ (%)
1	100	120	–	90	2
2	100	60	AcOH (2)	91	2
3	100	60	HCl (2)	4	29
4	100	60	H_2_SO_4_ (2)	3	40
5	100	60	MsCl (2)	4	50
6	100	15	TMSCl (2)	3	72
7	60	60	TMSCl (2)	<1	76
8^c^	50	60	TMSCl (1)	<1	75

a Using crude **2** reaction mixture (Table 1, entry 7).

b HPLC or GC calibrated yield against biphenyl as internal standard.

c Using crude **2** from Table 1, entry 9 (0.1 equiv NEt_3_).

The reaction producing **3** was easily scaled up to the gram scale; however, following aqueous wash and evaporation to dryness, surprisingly low yields were obtained. To determine in which step of the workup the product was lost, a mass balance was made by an HPLC assay after each step of the workup (Supporting Information, Table S5). It was clear that the majority of product was lost in the final workup step: solvent evaporation to dryness. This could be explained by the high volatility of the product, which evaporated at the employed pressure and temperature. By exploiting this behavior of compound **3**, a clean product could be obtained as a colorless crystalline solid by sublimation (37% yield, 60 °C, 15 mbar, Supporting Information, Figure S5).

To obtain a representative view of reaction performance, avoiding yield loss by evaporation, we assessed the amount of product in a concentrated EtOAc solution by an NMR assay (68% after workup, Supporting Information, Figure S4). Since product **3** would most likely be used as a starting material for onward reactions, it was deemed sufficient to obtain the product in solution, where it was found to be stable for extended time periods.

To prove the utility of product **3** for further modification and reach a more suitable point of isolation, we performed the two-step reaction to amidine hydrochloride salt **5** directly from the crude solution of **3** (Scheme 3). Since EtOAc was not a suitable solvent for the reaction with sodium methoxide (NaOMe), a solvent swap to methanol (MeOH) was performed without reducing the product to complete dryness at any point. After the addition of catalytic NaOMe (0.05 equiv), full conversion of **3** to benzimidate **4** was observed after 3 h at 0 °C.

**Scheme 3:**
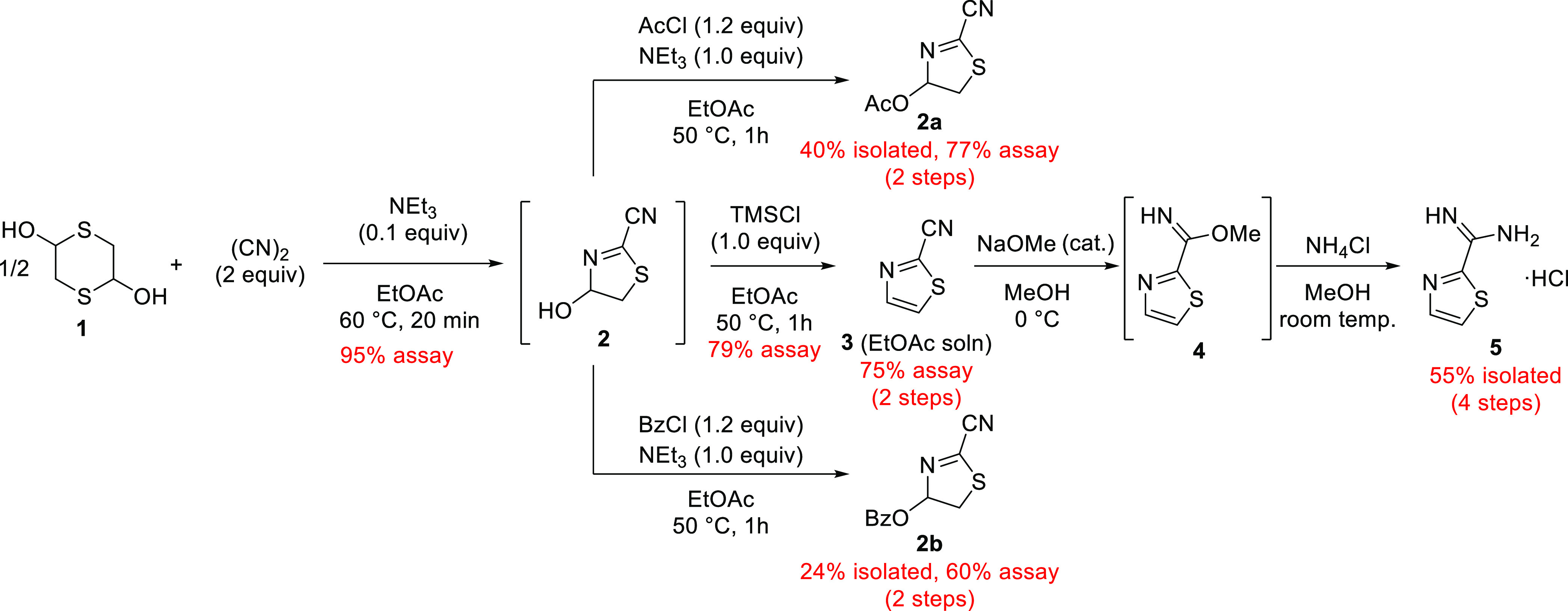
Four-Step Reaction Pathway from **2** to **5** Showing Alternative Functionalization Possibilities of **2** to **2a** and **2b**

Subsequently, ammonium chloride (NH_4_Cl) was added and the reaction was stirred at room temperature overnight. This procedure facilitated the straightforward isolation of **5**, by precipitation as the hydrochloride salt, providing an 83% yield for the individual step. Gratifyingly, the entire synthetic sequence could be carried out in 55% yield, over 4 steps, from bulk chemical starting materials dithiane **1** and (CN)_2_.

The intermediate **2** was proposed to be of interest from both synthetic and medicinal chemistry standpoints. There has been significant recent interest in the use of saturated heterocyclic linker molecules to increase solubility and explore new exit vectors.^21^ Since intermediate **2** could not be isolated, we attempted further functionalization from the crude reaction mixture. Simple acetylation of OH provided isolable product **2a** (Scheme 3). After aqueous basic washing with sodium bicarbonate, **2a** was produced in 77% assay yield and could be isolated in 40% yield (over two steps, from **1**).

To demonstrate that aromatic aroyl chlorides would also be amenable to this transformation, benzoylation of **2** using benzoyl chloride (BzCl) led to the formation of benzoylated product **2b**. This product was formed in 60% assay yield (over two steps from **1**). The pure compound was obtained as a white solid after column chromatography (24% isolated yield), demonstrating the proof-of-concept for this reactivity. It is envisaged that novel intermediate **2** will prove to be an interesting linker molecule for medicinal chemistry applications due to its unusual partial saturation and two functional handles.

In conclusion, we have achieved an atom-efficient and commercially attractive route to 2-cyanothiazole **3** via previously undescribed intermediate **2**. Furthermore, we showed the versatility of intermediate **2** by showing the acylation reactions to **2a** and **2b**, as well as conversion to amidine **5**. By revisiting an old method to generate (CN)_2_ gas, we demonstrated its effective use in a simple, yet effective, lab-scale setup. We anticipate that this will inspire further synthetic utilization of (CN)_2_, to exploit its untapped potential. The use of (CN)_2_ is expected to be more straightforward for large scale processing due to its large-scale commercial availability as a grain fumigant.

## Data Availability

The data underlying this study are available in the published article and its Supporting Information.

## Supporting Information Available

Further details of reaction setups, experimental results and NMR data (PDF)
