# Supplementary material for: Rediscovering Cyanogen Gas for Organic Synthesis: Formation of 2-Cyanothiazole Derivatives
Source: J Org Chem. 2023 Jun 20;88(13):9594–8. doi: 10.1021/acs.joc.3c01110 (PMC10337028; doi:10.1021/acs.joc.3c01110)
Supplement: Supplementary file 1 — jo3c01110_si_001.pdf [file jo3c01110_si_001.pdf]

Supporting Information

Rediscovering Cyanogen Gas for Organic Synthesis: Formation of 2-Cyanothiazole Derivatives

Michael Prieschl,[†,‡] Joerg Sedelmeier,[§] Kurt Püntener,[§] Stefan Hildbrand,[§] Jason D. Williams,*[,†,‡] C. Oliver Kappe*[,†,‡]

[†]Center for Continuous Flow Synthesis and Processing (CC FLOW), Research Center Pharmaceutical Engineering GmbH (RCPE), Inffeldgasse 13, 8010 Graz, Austria

[‡]Institute of Chemistry, University of Graz, NAWI Graz, Heinrichstrasse 28, 8010 Graz, Austria

[§]Department of Process Chemistry & Catalysis, F. Hoffmann-La Roche Ltd, 4070 Basel, Switzerland

Contents

1. Materials and Methods S3
2. Experimental Procedures S5
   2.1 General Procedure - Synthesis of 4-hydroxy-4,5-dihydrothiazole-2-carbonitrile S5
   2.2 Synthesis of 4-Hydroxy-4,5-dihydrothiazole-2-carbonitrile – Scale-up S7
   2.3 Synthesis of 2-Cyano-4,5-dihydrothiazol-5-yl Acetate S8
   2.4 Synthesis of 2-Cyano-4,5-dihydrothiazol-5-yl Benzoate S8
   2.5 Synthesis of 2-Cyanothiazole – General Procedure S9
   2.6 Synthesis of 2-Cyanothiazole – Scale-up S9
   2.7 Synthesis of Thiazole-2-carboximidamide Hydrochloride S10
   2.8 Synthesis of Thiazole-2-carboximidamide Hydrochloride – Telescoped Procedure S10
3. Additional Experimental Results S11
   3.1 Model Reaction with Cysteamine S11
   3.2 $(CN)_2$ Addition to 1,4-Dithiane-2,5-diol S12
   3.3 Dehydration Reaction to 2-Cyanothiazole S13
   3.4 Isolation of 2-Cyanothiazole S14
   3.5 Identification of Side Products S15
   3.6 Determination of Assay Yield for Crude 3 .... S17
   3.7 Purification of Product 3 by Sublimation S17
   3.8 Calibration Data S18
      3.8.1 GC Calibration S18
      3.8.2 HPLC Calibration S18
4. Analytical Data S19
   4.1 4-Hydroxy-4,5-dihydrothiazole-2-carbonitrile (2) S19
   4.2 2-Cyano-4,5-dihydrothiazol-5-yl Acetate (2a) S21
   4.3 2-Cyano-4,5-dihydrothiazol-5-yl Benzoate (2b) S23
   4.4 Synthesis of 2-Cyanothiazole (3) S25
   4.5 Thiazole-2-carboximidamide hydrochloride (5) S27
5. References S29

1. Materials and Methods

Caution: Dangers of Cyanogen Due to the high toxicity of $(CN)_2$, care must be taken to ensure that all generated $(CN)_2$ is contained and any unreacted gas is quenched by oxidation in a basic environment.[1] It is likely that HCN is also formed during the process, which requires additional caution.[2] Chemistry should only be performed in a well-ventilated fume hood, after performing a proper risk assessment, which considers the fate of all liquid and gaseous streams. Further information about the toxicological properties of $(CN)_2$, including a comparison of hazards with HCN, can be found in literature.[3]

Small scale **heated reactions** were heated using magnetic stirrer hotplate with a stainless steel heating mantle. Larger scale experiments were heated using a thermostated water bath on a magnetic stirrer hotplate.

^{1}H- and ^{13}C NMR spectra were recorded on a Bruker Avance III 300 MHz instrument at ambient temperature, in $CDCl_3$ or DMSO-d_6 as solvent, at 300 MHz and 75 MHz, respectively. Chemical shifts (δ) are reported in ppm using TMS as internal standard. Coupling constants are given in Hz units. The letters s, d, t, q, and m are used to indicate singlet, doublet, triplet, quartet, and multiplet, respectively.

Analytical **HPLC analysis** was carried out on a C18 reversed-phase (RP) analytical column (150 × 4.6 mm, particle size 5 mm) at 37 °C by using mobile phases A [water/acetonitrile 90:10 (v/v) + 0.1% TFA] and B (acetonitrile + 0.1% TFA) at a flow rate of 1.5 mL/min.

The following gradient was applied: Linear increase from 3% solution B to 5% B over 3 min, linear increase from 5% B to 30% B over 4 min, linear increase from 30% B to 100% B over 3 min, hold at 100% B for 2 min, linear decrease from 100% B to 3% B over 0.5 min, hold at 3% B for 2.5 min.

GC-FID analysis was performed on a Shimadzu GC FID 230 with a flame ionization detector, using an RTX-5MS column (30 m × 0.25 mm ID × 0.25 µm) and helium as carrier gas (40 cm sec^{-1} linear velocity). The injector temperature was set to 280 °C. After 1 min at 50 °C, the temperature was increased by 25 °C/min to 300 °C and kept constant at 300 °C for 4 min. The detector gases used for flame ionization were hydrogen and synthetic air (5.0 quality).

LC-MS analysis was carried out on a Shimadzu instrument using a C18 reversed-phase (RP) analytical column (150 mm × 4.6 mm, particle size 5 µm using mobile phases A (H_2O/MeCN 90:10 (v/v) + 0.1% HCOOH) and B (MeCN + 0.1 % HCOOH) at a flow rate of 0.6 mL/min. The following gradient was applied: hold at 5% solvent B until 2 min, increase to 20% solvent B until 8 min, increase to 100% solvent B until 16 min and hold until 22 min at 100% solvent B. Low resolution mass spectra were obtained on a Shimadzu LCMS-QP2020 instrument using electrospray ionization (ESI) in positive or negative mode.

High-resolution mass spectrometry (**HRMS**):

Method A: Measurements were carried out with an Agilent 6230 TOF mass spectrometer, after injection on an Agilent 1260 Infinity Series HPLC system. The injection volume was set to 0.5 µL and the flow rate to 0.3 mL/min of a mixture of 40% H_2O (0.1% 5 M ammonium formate) and 60% MeCN/H_2O (5:1+0.1% 5 M ammonium formate). The HRMS module comprises an electrospray ionization source (Dual AJS ESI) and uses nitrogen as the nebulizer (15 psig) and the drying gas (5 L/min). ESI experiments were performed using the positive ionization mode (Gas Temp. = 300 °C, Fragmentor = 150 V, Skimmer = 65 V, OCT 1 RF Vpp = 750 V,Vcap = 1400, Nozzle Voltage = 2000 V, Reference Masses = 121.050873 and 922.009798, Acquisition = 100-1100 m/z, 1 spectra/s).

Method B: Measurements were carried out with an Agilent 6546qTOF mass spectrometer with a dual Jet Stream Technology Ion Source (AJS), after injection on an Agilent 1290 UHPLC system with a cooled autosampler. The column used was a Zorbax SB-C18 (2.1 + 50 mm, 1.8 µm) from Agilent. As solvents 0.1 % (v/v) formic acid in water (channel A) respectively in methanol (channel B) were used.

The gradient was from 0 % B linear to 100 % B in 10 min, hold for 5 min and then re-equilibration of the column. The flow rate was 0.4 mL/min with a sample volume of 10 µL and a column temperature of 35°C. The HRMS instrument was run in positive scan mode from 100 to 500 m/z with continuous infusion of an internal standard solution containing among others caffeine. Capillary voltage applied was 4.5 kV, gas temperature 150°C (flow 6 L/min), nebulizer pressure 30 psi, and sheath gas temperature 350°C (flow 10 L/min), fragmentor voltage 110 V. For MSMS experiments the collision energy was set to 10.

Infrared spectra (**IR**): measured on a Bruker alpha p instrument, using attenuated total reflectance (ATR). Spectra were processed using OPUS v6.5 software. The suffixes br, s, m and w denote a broad, strong, medium or weak signal, respectively.

Melting point measurements were performed using a Stuart SMP3 melting point apparatus.

2. Experimental Procedures

2.1 General Procedure - Synthesis of 4-hydroxy-4,5-dihydrothiazole-2-carbonitrile

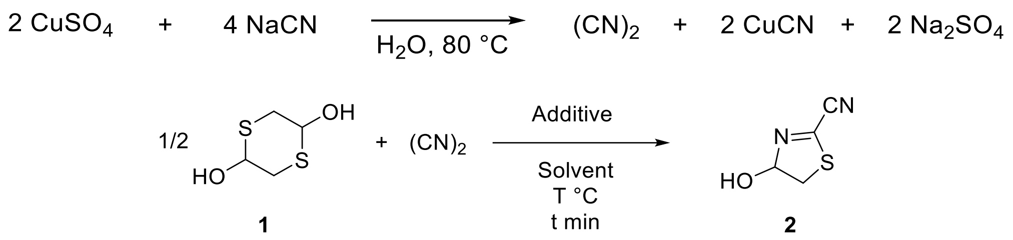

NaCN solution (aqueous):

NaCN was added to a volumetric flask. The NaCN was dissolved in water and brought up to a final volume with H_2O to form a solution with 4 M concentration.

Solution A:

$CuSO_4$ (2.0 g, 8.0 mmol) was placed in a round bottom flask and dissolved in water (4 mL) at 80 °C to form a deep blue solution at a target concentration of 2 M.

Solution B:

1,4-dithiane-2,5-diol (152 mg, 1 mmol) was placed in a round-bottom flask and suspended in EtOAc (10 mL) to a final concentration of 0.1 M. Additive was added to the white suspension. Solution B was heated to 60 °C.

Procedure:

The flask containing solution A was connected to solution B using needles, a short piece of PFA tubing (1/16 inch) and suitable adapters to transfer the evolved gas. The flask containing solution B was connected to a quench solution containing basic bleach solution ($NaHCO_3$/ NaOH buffer + 1 M NaOCl solution) to consume excess $(CN)_2$. See Figure S1 for a photograph of the reaction setup.

The NaCN solution was added to solution A over 5 min using a dropping funnel or syringe pump. Heavy bubbling and formation of colorless solid was observed. After the addition of NaCN was finished solution B formed a colorless solution. Solution B was stirred for a further 15 min (slight off coloring observed over time). Compound **2** was obtained in as a solution in EtOAc.

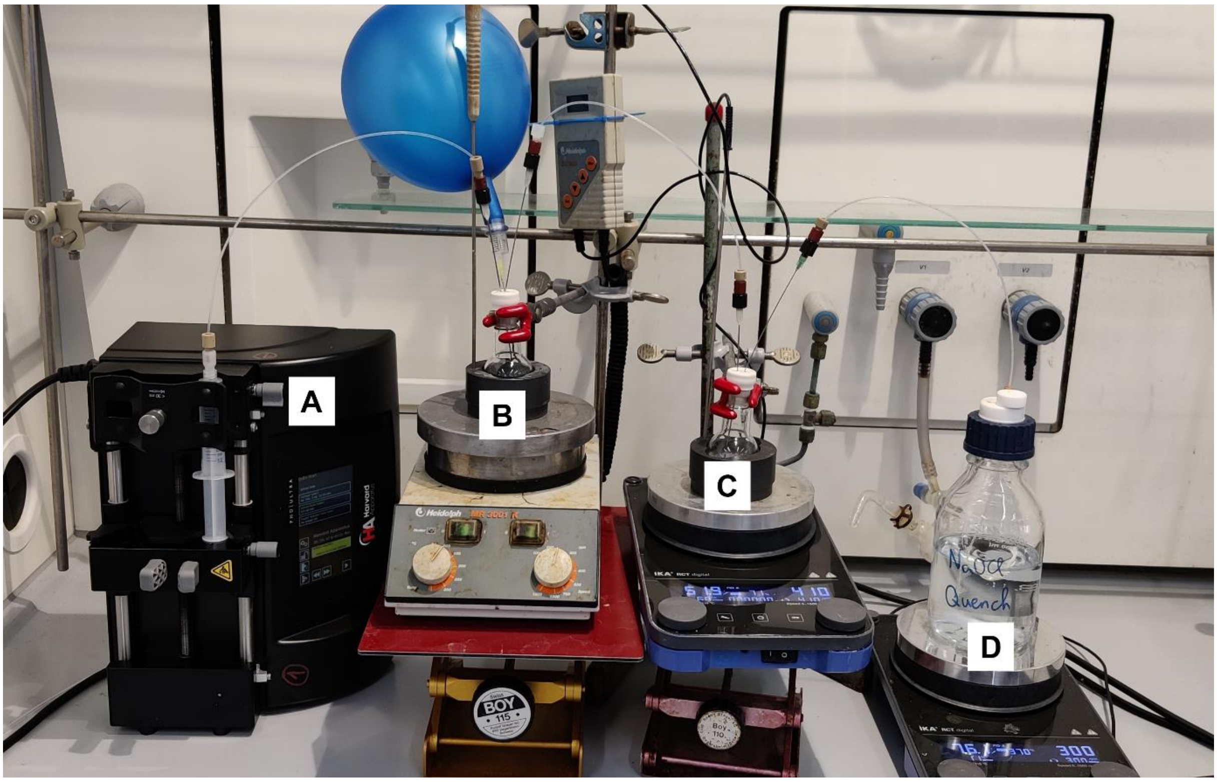

Figure S1. Setup for the formation of **2**. **A**: Syringe pump (Harvard, PHD Ultra) for the delivery of NaCN solution. **B**: Flask containing $CuSO_4$ solution for $(CN)_2$ formation. The evolved gas was transferred to **C** via PFA tubing (0.8 mm i.d.) and needles (with the appropriate adapters) under pressure of argon from an argon-filled balloon. **C**: Flask containing solution of **1** for the formation of **2** with $(CN)_2$. **D**: Aqueous basic (pH ~ 10) solution of NaOCl for the quench of excess $(CN)_2$.

2.2 Synthesis of 4-Hydroxy-4,5-dihydrothiazole-2-carbonitrile – Scale-up

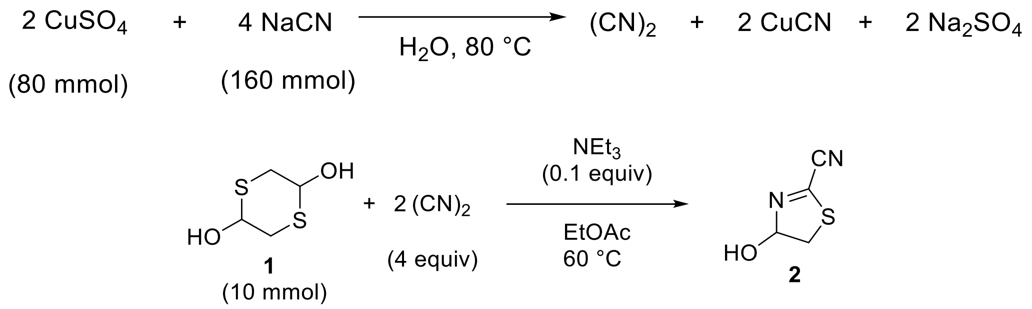

4 M NaCN solution (aqueous):

A NaCN (9.80 g, 200 mmol) was added to a 50 mL volumetric flask. The NaCN was dissolved in water and brought up to a final volume of 50 mL with H_2O.

Solution A:

$CuSO_4$ (19.97 g, 80 mmol) was placed in a 250 mL three neck round bottom flask and dissolved in water (40 mL) at 80 °C to form a deep blue solution.

Solution B:

1,4-dithiane-2,5-diol **1** (1.52 g, 10 mmol) was placed in a 250 mL two neck flask and suspended in EtOAc (100 ml). Triethylamine (139 µL, 1 mmol, 0.1 equiv) was added to the white suspension. Solution B was heated to 60 °C.

Procedure:

The flask containing solution A was connected to solution B using needles, a short piece of PFA tubing (1/16 inch) and suitable adapters to transfer the evolved gas. The flask containing solution B was connected to a quench solution containing basic bleach solution ($NaHCO_3$/ NaOH buffer + 1 M NaOCl solution) to consume excess $(CN)_2$.

The 4 M NaCN solution was added to solution A over 5 min using a dropping funnel. Heavy bubbling and formation of colorless solid was observed. After the addition of NaCN was finished (theoretical $(CN)_2$ quantity: 40 mmol, 4 equiv) solution B cleared up and a colorless solution was formed. Solution B was stirred for a further 15 min (slight off coloring observed over time). The title product **2** was obtained in ~91-96% HPLC assay yield, as a solution in EtOAc.

2.3 Synthesis of 2-Cyano-4,5-dihydrothiazol-5-yl Acetate

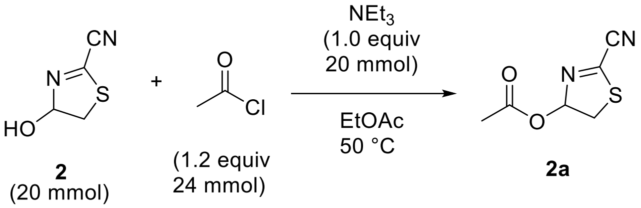

2 (20 mmol) (1.2 equiv 24 mmol) **2a**

Triethylamine (2.79 mL, 20 mmol, 1.0 equiv) was added to the crude solution of **2** (prepared according to section 2.2) at 50 °C. A colorless suspension was formed. Acetyl chloride (1.71 mL, 24 mmol, 1.2 equiv) was added subsequently. After stirring for 1 h at 50 °C the reaction was quenched with water (100 mL).

The organic phase was separated and washed with $NaHCO_3$ (5% aqueous soln, 2 × 100 mL). The combined aqueous phases were back-extracted twice using EtOAc (2 × 50 mL). The combined organic phases were dried over Na_2SO_4 and filtered. The solution was reduced under vacuum to dryness. 3.14 g (93%) of crude product was obtained as a yellow to brown oil with 84% NMR purity (77% yield of pure compound).

Isolation experiment: Triethylamine (279 µL, 2 mmol, 1.0 equiv) was added to the crude solution of **2** (prepared according to section 2.1) at 50 °C. A colorless suspension was formed. Acetyl chloride (171 µL, 2.4 mmol, 1.2 equiv) was added subsequently. After stirring for 1 h at 50 °C the reaction was quenched with water (10 mL).

The organic phase was separated and washed with $NaHCO_3$ (5% aqueous soln, 2 × 10 mL). The combined aqueous phases were back-extracted twice using EtOAc (2 × 5 mL). The combined organic phases were dried over Na_2SO_4 and filtered. The was then purified by flash column chromatography (EtOAc:cyclohexane, 10%-50% EtOAc) to afford the title compound as a pale yellow oil (851 mg, 40% yield, >95% NMR purity).

2.4 Synthesis of 2-Cyano-4,5-dihydrothiazol-5-yl Benzoate

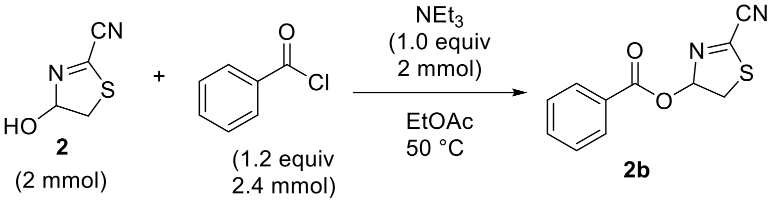

2 (2 mmol) (1.2 equiv 2.4 mmol) **2b**

Triethylamine (279 µL, 2 mmol, 1.0 equiv) was added to the crude solution of **2** (prepared according to section 2.1) at 50 °C. A colorless suspension was formed. Benzoyl chloride (461µL, 2.4 mmol, 1.2 equiv) was added subsequently. After stirring for 1 h at 50 °C the reaction was quenched with water (10 mL).

The organic phase was separated and washed with $NaHCO_3$ (5% aqueous soln, 2 × 10 mL). The combined aqueous phases were back-extracted twice using EtOAc (2 × 5 mL). The combined organic phases were dried over Na_2SO_4 and filtered. The solution was reduced under vacuum to dryness. 475 mg (104%) of crude product was obtained as brown oil with 58% purity (60% assay yield of pure compound). The product was then purified by flash column chromatography (EtOAc:cyclohexane, 0%-40% EtOAc) to afford the title compound as a white solid (110 mg, 24% yield, >95% NMR purity).

2.5 Synthesis of 2-Cyanothiazole – General Procedure

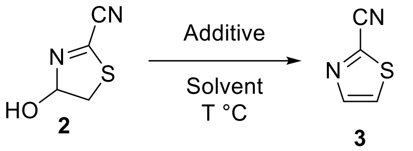

Additive was added to the crude solution of **2** (prepared according to section 2.1) at reaction temperature. Samples for analysis were diluted in MeCN and analyzed by GC/HPLC (samples for GC were neutralized and dried over Na_2SO_4). After stirring at reaction temperature, the reaction was quenched with water.

Workup:

The organic phase was separated and washed with $NaHCO_3$ (5% aqueous soln, 2 × 100 mL). The combined aqueous phases were back-extracted twice using EtOAc (2 × 50 mL). The combined organic phases were dried over Na_2SO_4 and filtered. The solution was reduced under vacuum to ~5% of the original volume.

2.6 Synthesis of 2-Cyanothiazole – Scale-up

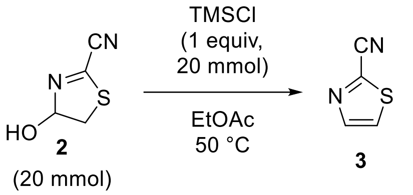

Trimethylsilyl chloride (2.54 mL, 20 mmol, 1 equiv) was added to the crude solution of **2** (prepared according to section 2.2) at 50 °C. After stirring for 1 h at 50 °C the reaction was quenched with water (100 mL). HPLC assay: 71-76% (over two steps)

The organic phase was separated and washed with $NaHCO_3$ (5% aqueous soln, 2 × 100 mL). The combined aqueous phases were back-extracted twice using EtOAc (2 × 50 mL).

The combined organic phases were dried over Na_2SO_4 and filtered. The solution was reduced under vacuum to ~5% of the original volume. The desired product was quantified as 67.5% NMR assay yield (over two steps) against 1,3,5-trimethoxybenzene as standard.

The desired product was purified by sublimation at 60 °C and 15 mbar pressure, to afford a white crystalline solid (835 mg, 37% yield).

2.7 Synthesis of Thiazole-2-carboximidamide Hydrochloride

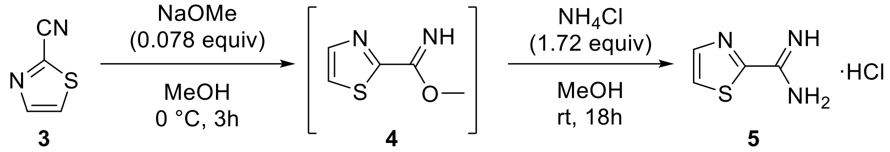

Crude **3** was added to a 25 mL round bottom flask (6.4 mmol, 1.1 g crude with 62% purity, 1 equiv) and dissolved in 6 mL of MeOH. The solution was cooled to 0 °C and NaOMe (25 wt% in MeOH, 0.5 mmol, 114 µL, 0.078 equiv) was added, forming a brown suspension. After stirring for ~3 h the reaction was allowed to warm to room temperature (crude solution of intermediate **4**).

Ammonium chloride (11 mmol, 588 mg, 1.72 equiv) was added to the mixture. The resulting mixture was stirred for ~18 h at room temperature. Solids were filtered off over celite, washed with MeOH and disposed. Solvent (MeOH) was replaced by acetonitrile (four times 5 mL portions of MeCN added, with evaporation to ~10 mL volume). Solid formation was observed when acetonitrile was added to the mixture. Water (500 µL) was added to the suspension, which was then heated to 85 °C for 1 h. A dark brown solution was formed. The solution was stirred at room temperature for ~3 h. Solid formation was observed. The suspension was then cooled to 0 °C for ~1 h. Large amounts of brown solid were formed.

The brown solid was isolated by filtration and washed with acetonitrile. 1.10 g of crude material was collected (103 %). The solid was dried for ~16 h at 50 °C under vacuum. 898 mg of brown solid product was obtained after drying (898 mg, 88% yield, 95% NMR assay purity). This corresponds to 83% yield of pure product **5** over two steps

2.8 Synthesis of Thiazole-2-carboximidamide Hydrochloride – Telescoped Procedure

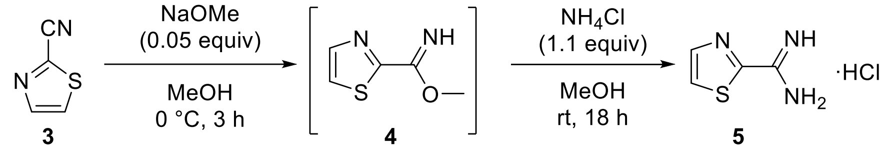

Crude EtOAc solution of **3** (prepared according to the procedure from section 2.6 – not concentrated to dryness). A solvent swap was performed from EtOAc to MeOH (10 mL final volume).

The solution was cooled to 0 °C and NaOMe (25wt% in MeOH, 1.0 mmol, 229 µL, 0.05 equiv) was added, forming a brown suspension. After stirring for ~3 h the reaction was allowed to warm to room temperature (crude solution of intermediate **4**).

Ammonium chloride (22 mmol, 1.18 mg, 1.1 equiv) was added to the mixture. The resulting mixture was stirred for ~18 h at room temperature. Solids were filtered off, washed with MeOH and disposed. Solvent (MeOH) was replaced by acetonitrile (four times 5 mL portions of MeCN added, evaporation to ~10 mL volume). Solid formation was observed when acetonitrile was added to the mixture. Water (1 mL) was added to the suspension.

The slight brown solid was isolated by filtration and washed with acetonitrile and 40-60 petroleum ether. 1.40 g of material was collected. The mother liquor was reduced in volume until a large amount of solid formation was observed. The solid was isolated by filtration and washed with MeCN and 40-60 petroleum ether. Product **5** (1.8 g, 55% yield over 4 steps, 99% NMR assay purity) was obtained as a brown crystalline solid.

3. Additional Experimental Results

3.1 Model Reaction with Cysteamine

Table S1. Experiments with cysteamine as a model substrate to evaluate $(CN)_2$ formation efficiency

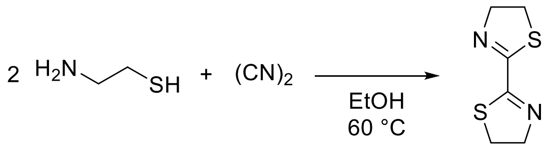

Entry[a]	Limiting reagent	CN_2 equiv	Reaction Temp[b] (°C)	Conv Cysteamine (%)[c]
1[d]	$CuSO_4$	1.0	-40	9%
2	NaCN	1.0	-40	22%
3	NaCN	2.0	60	82%

[a]$(CN)_2$ generated over ~5 min, reaction for 1 h at 60 °C, 2 M $CuSO_4$ soln. (aq.), 4 M NaCN soln. (aq.). [b]Temperature of the reaction flask at the time of $(CN)_2$ addition. All reactions were then warmed to 60 °C for reaction. [c]Calculated from GC analysis vs biphenyl as internal standard. [d]1 M $CuSO_4$ soln. and 2 M NaCN soln.

3.2 $(CN)_2$ Addition to 1,4-Dithiane-2,5-diol

Table S2. Optimization of 1,4-dithiane-2,5-diol **1** reaction with $(CN)_2$

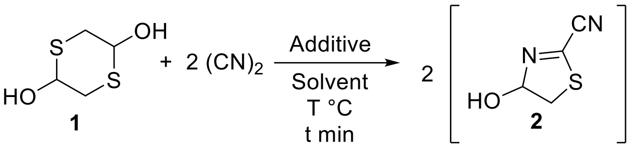

Entry[a]	Solvent	T (°C)	Time (min)[b]	Additive (equiv)	Equiv $(CN)_2$[c]	**2** (%)[d]
1	EtOH	60	5	DIPEA (1.0)	2.0	12
2	EtOH	60	15	DIPEA (1.0)	2.0	11
3	EtOH	0	60	DIPEA (1.0)	2.0	11
4	EtOH	30	15	DIPEA (1.0)	8.0	39
5	EtOH	30	15	DIPEA (1.0)	16.0	53
6	EtOH	30	15	HCl (1.0)	4.0	9
7	CF_3CH_2OH	30	15	DIPEA (1.0)	4.0	20
8	iPrOAc	30	0	DIPEA (1.0)	4.0	58
9	EtOAc	30	0	DIPEA (1.0)	4.0	73
10	EtOAc	30	10	DIPEA (1.0)	4.0	97
11	EtOAc	30	30	DIPEA (1.0)	8.0	93
12	EtOAc	30	30	HCl (1.0)	4.0	44
13	EtOAc	30	0	DIPEA (1.0)	4.0	45
14	EtOAc	30	0	-	4.0	53
15	EtOAc	30	15	DBU (1.0)	4.0	3
16	EtOAc	30	15	DABCO (1.0)	4.0	22
17	EtOAc	30	15	TMG (1.0)	4.0	13
18	EtOAc	30	15	DIPEA + TMSCl (1.0)	4.0	74
19[e]	EtOAc	60	0	DIPEA (1.0)	4.0	88
20[e]	EtOAc	30	0	DIPEA (1.0)	4.0	76
21	EtOAc	60	15	TEA (0.5)	4.0	92

[a]0.1 M concentration of **1**. [b]Time after dosing of $(CN)_2$ was finished. [c]Assuming 100% yield of $(CN)_2$ generation reaction. [d]HPLC or GC calibrated yield against biphenyl as internal standard. [e]0.2 M concentration of **2**.

Table S3. Optimization of $(CN)_2$ addition time (controlling addition of NaCN to $CuSO_4$)

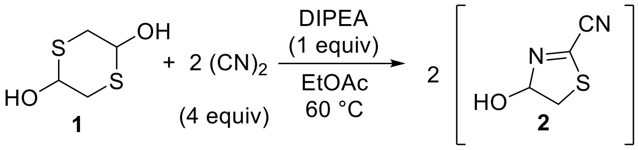

Entry[a]	Time (min)[b]	$t_{addition}$ $(CN)_2$ (min)[c]	**2** (%)[d]
1	5	1	88
2	15	5	92
3	15	15	80

[a]0.1 M concentration of **1** in EtOAc, 4.0 equiv of $(CN)_2$, 60 °C reaction temperature, 1 equivalent of DIPEA. [b]Time after dosing of $(CN)_2$ was finished. [c]Addition time of NaCN for the generation of $(CN)_2$, which was controlled by a syringe pump (Harvard, PHD Ultra). [d]HPLC calibrated yield against biphenyl as internal standard.

3.3 Dehydration Reaction to 2-Cyanothiazole

Table S4. Optimization of dehydration reaction of **2** to **3**

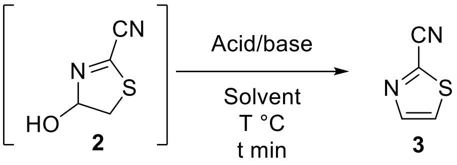

Entry[a]	Solvent	T (°C)	Time (min)	Acid/base (equiv)	**2** (%)[b]	**3** (%)[b]
1	EtOH	100	60	-	10	15
2	EtOH	100	60	HCl (1.0)	7	4
3	EtOH	100	60	HCl (2.0)	7	4
4	EtOAc	60	120	-	94	2
5	EtOAc	100	60	HCl (1.0)	<1	40
6	EtOAc	60	60	HCl (1.0)	<1	36
7	EtOAc	60	60	HCl (2.0)	<1	25
8	EtOAc	100	60	pTSA (2.0)	3	31
9	EtOAc	50	15	$ZnCl_2$ (1.0)	<1	17
10	EtOAc	50	15	$ZnCl_2$ (2.0)	<1	11
11	EtOAc	100	60	DBU (2.0)	<1	7
12	EtOAc	100	60	TMG (2.0)	<1	2
13	EtOAc	100	60	DABCO (2.0)	41	15
14	EtOAc	100	60	NaOH (2.0)	<1	1
15	EtOAc	rt	180	TMSCl (1.0)	53	6
16	EtOAc	60	120	TMSCl (0.5)	2	67
17	EtOAc	60	60	TMSCl (1.5)	<1	73
18	EtOAc	60	60	TMSCl (3.0)	<1	76

[a]Crude reaction mixture containing **2** was used as reaction input. [b]HPLC or GC calibrated yield against biphenyl as internal standard.

3.4 Isolation of 2-Cyanothiazole

Table S5. Mass balance of product **3** for each step of the workup

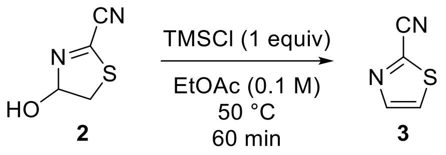

Workup step	Yield **3** (%)[a]
Before Workup	75
After H_2O quench	72
After aqueous washes	72
Evaporation to ~10% volume	75
Evaporation to dryness	61

[a]HPLC assay against biphenyl as internal standard.

3.5 Identification of Side Products

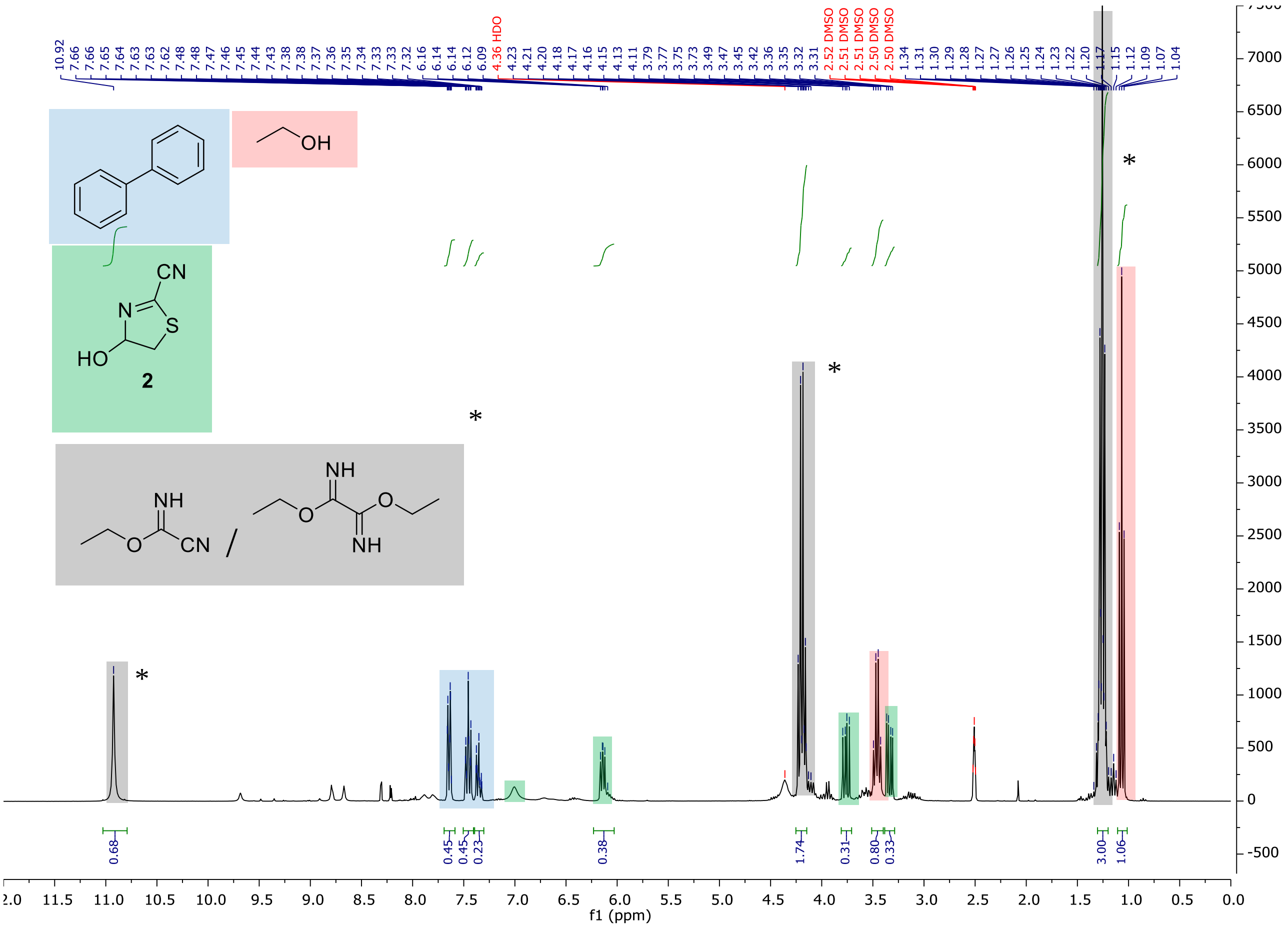

Figure S2. ^{1}H NMR spectrum of crude reaction mixture (after concentration by evaporating EtOH) after adding $(CN)_2$ to **1** in EtOH as reaction solvent. Major peaks are identified by color-coding. Asterisks (*, marked in gray) mark peaks belonging to a side product that is most likely formed by addition of EtOH to $(CN)_2$. The singlet at 10.92 ppm is thought to correspond to an N-H, whilst the quartet at ~4.2 ppm and the triplet at ~1.2 ppm correspond to a CH_2-CH_3 group.

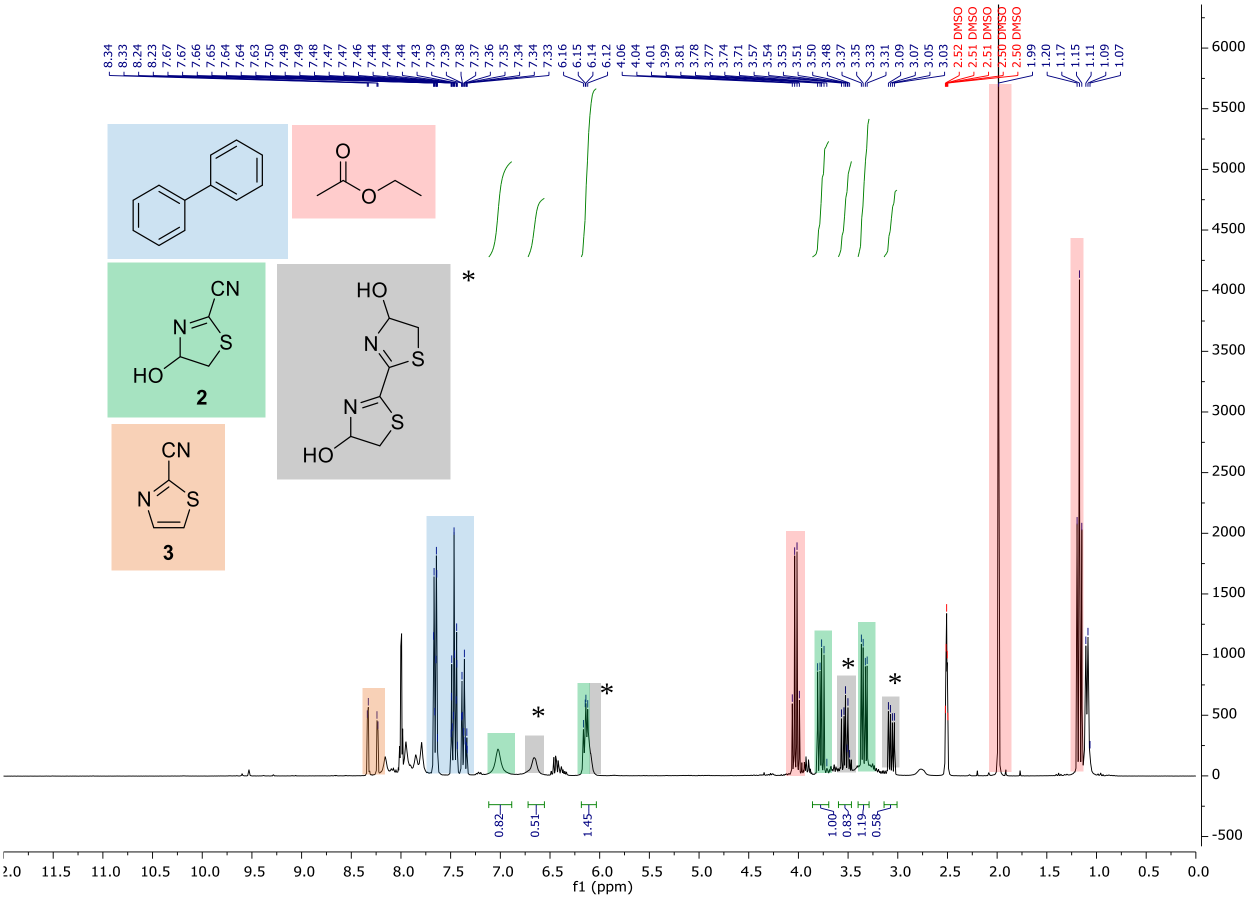

Figure S3. ^{1}H NMR spectrum of crude **2** after reaction in EtOAc and evaporation of solvent until dryness is almost reached. Besides EtOAc and product **2**, the mixture also shows peaks belonging to product **3**, which most likely forms when the concentration of the solution increases. Peaks marked with an asterisk (*, marked in gray) seem to belong to a pseudo-dimer of **2**, which could be formed by reaction of **2** with another equivalent of 2-mercaptoacetaldehyde at higher concentration.

3.6 Determination of Assay Yield for Crude 3

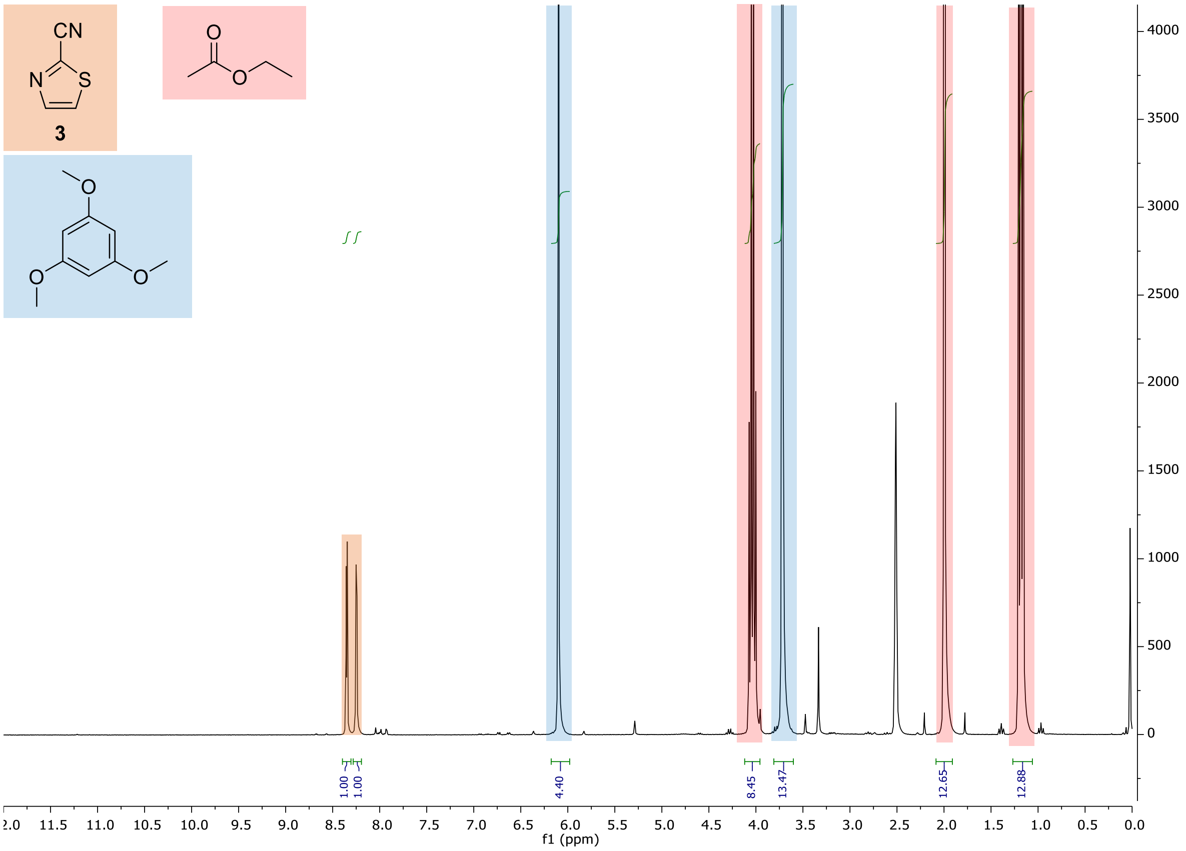

Figure S4. ^{1}H NMR spectrum of **3** after reaction in EtOAc and aqueous wash. Solvent (red) was not evaporated to dryness to avoid evaporation of product **3** (orange). NMR yield was determined against 1,3,5-trimethoxybenzene (blue).

3.7 Purification of Product 3 by Sublimation

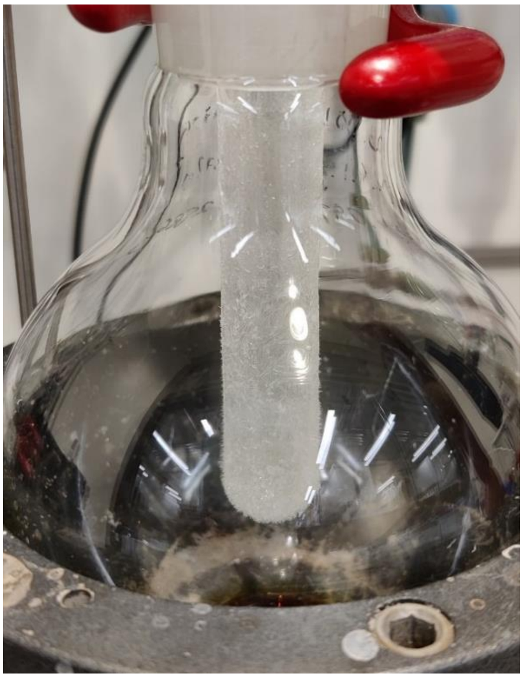

Figure S5. Photograph of product **3** sublimation at 60°C and 15 mbar.

3.8 Calibration Data

3.8.1 GC Calibration

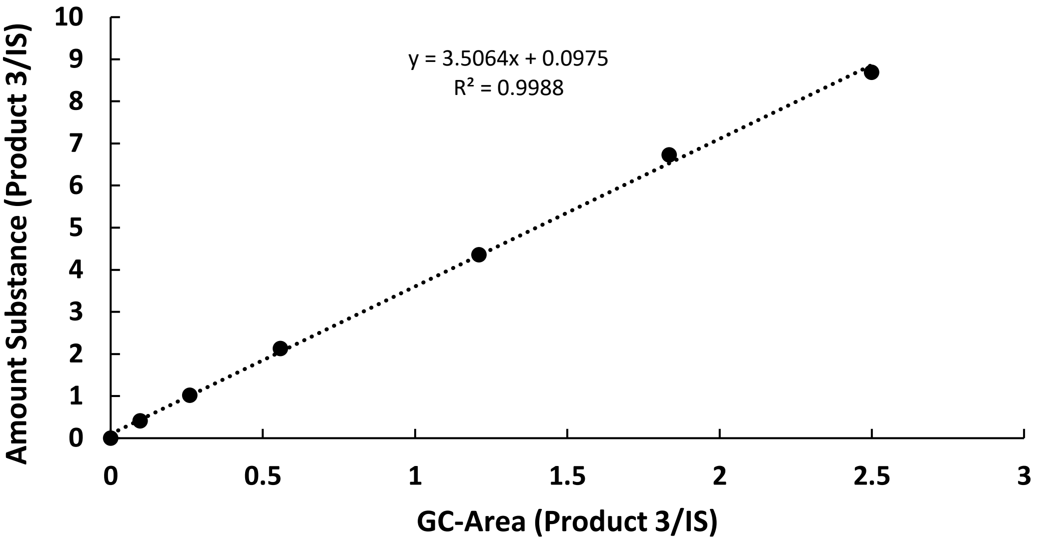

Figure S6. GC calibration curve of product **3** against biphenyl as internal standard.

For the determination of GC assay yield for product **2**, the same calibration curve was used, since **2** could not be isolated. Because the calibration for product **3** underestimated the amount of **2** that was actually present, a correction was performed by NMR based on 5 reaction samples. Through comparison of the values, a correction factor of 1.49 was found. By multiplication of the GC assay yields with the correction factor a corrected GC assay for **2** could be determined.

3.8.2 HPLC Calibration

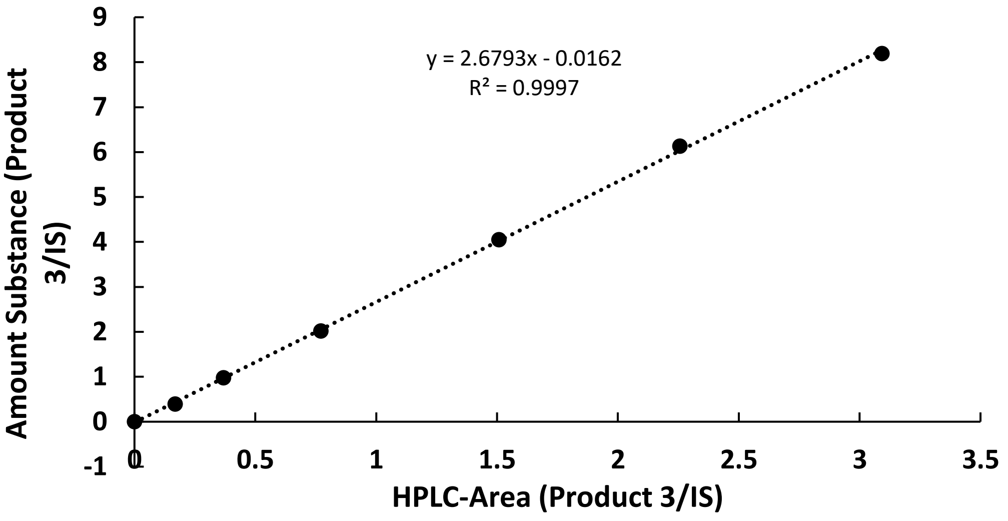

Figure S7. HPLC calibration curve of product **3** against biphenyl as internal standard (254 nm wavelength).

By using the same procedure NMR assay procedure, a correction factor of 5.83 was determined for HPLC assay of **2** (254 nm wavelength).

4. Analytical Data

4.1 4-Hydroxy-4,5-dihydrothiazole-2-carbonitrile (2)

Decomposition was observed when concentrating samples under vacuum at 40 °C. Crude analysis data is reported.

HPLC Analysis

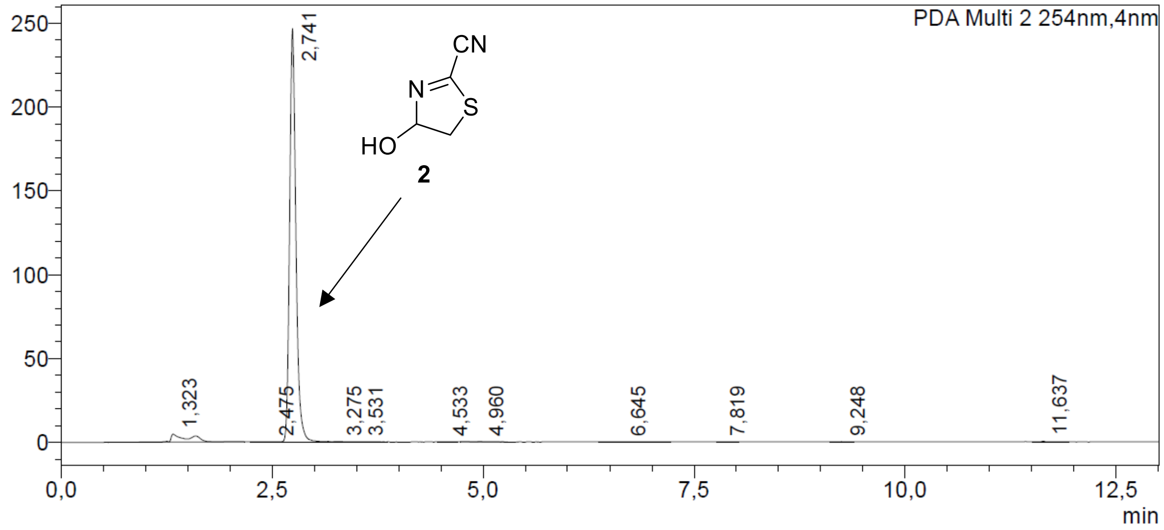

HPLC trace of the crude reaction mixture following the procedure from section 2.2. The HPLC sample was taken 15 minutes after the addition of NaCN (for $(CN)_2$ formation) was finished.

Retention Time: 2.741 min

HPLC Purity: 94.0%

HRMS

Method A: m/z$[M+H]^+$ calcd. for $[C_4H_5N_2OS]$: 129.0123 found: 129.0112

NMR Spectroscopy

^{1}H NMR (300 MHz, DMSO-d_6) δ 6.99 (d, *J* = 7.0 Hz, 1H), 6.14 (dd, *J* = 13.0, 5.3 Hz, 1H), 3.78 (dd, *J* = 12.0, 7.4 Hz, 2H), 3.34 (dd, *J* = 12.1, 5.5 Hz, 1H).

^{1}H NMR Spectrum (crude)

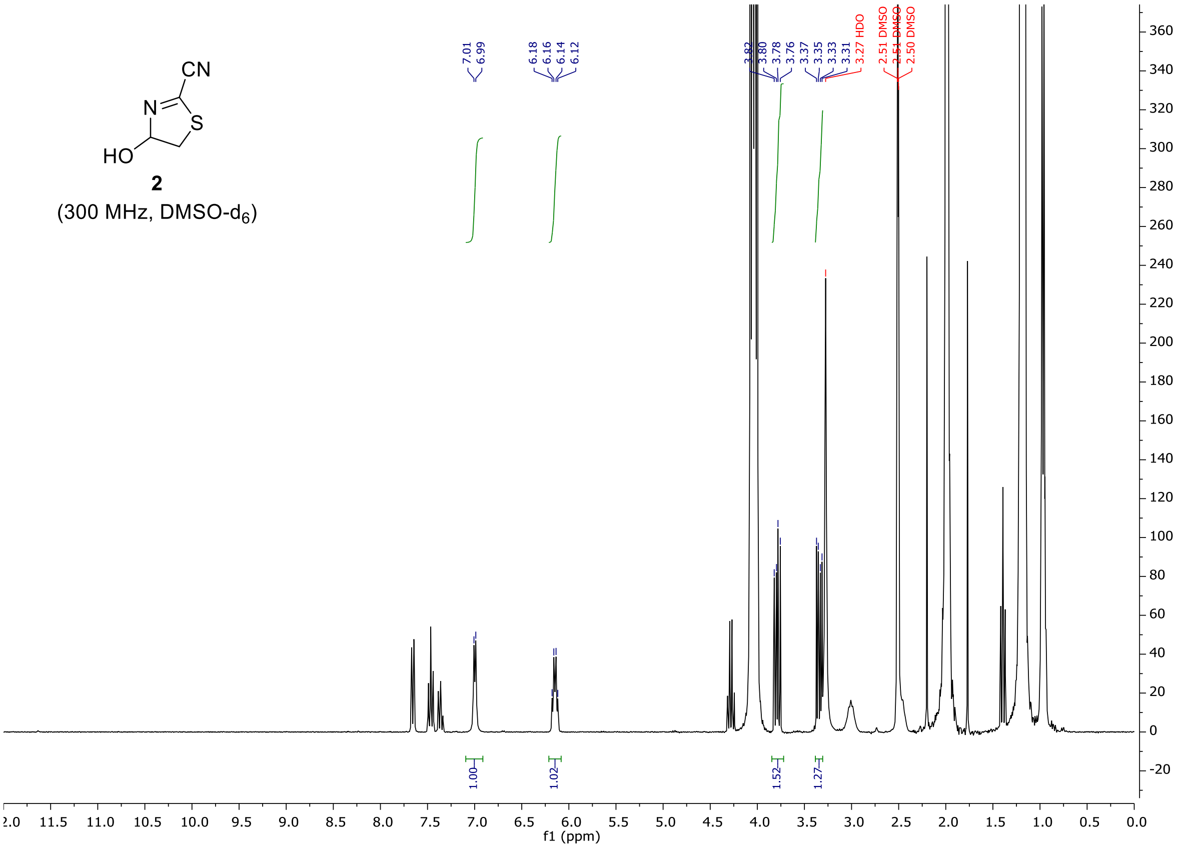

^{1}H NMR spectrum (crude) obtained by diluting 200 µL of crude reaction mixture (sample taken 15 min after $(CN)_2$ formation was finished) with 400 µL of DMSO-d_6. Peaks belonging to the desired product are marked and integrated. The diastereotopic protons at ~3.3-3.8 ppm overlap in part with peaks belonging to solvent (^{13}C satellite of EtOAc CH_2 group) and H_2O.

4.2 2-Cyano-4,5-dihydrothiazol-5-yl Acetate (2a)

HPLC Analysis

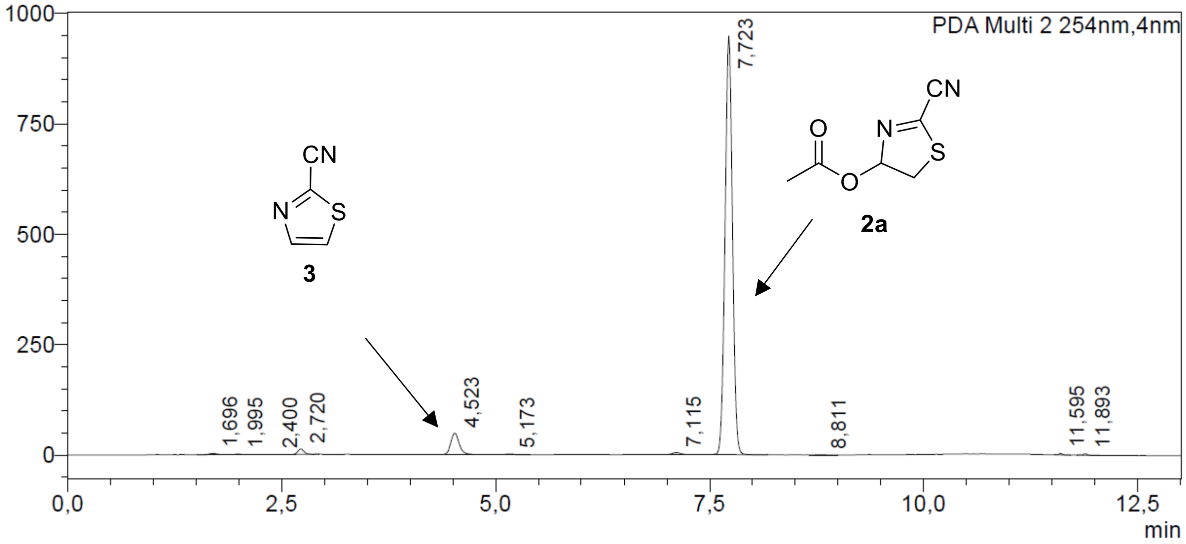

HPLC trace after column chromatography.

Retention Time: 7.723 min

HPLC Purity: 92.3% area (content of **3** is over-represented due to far higher absorbance at 254 nm)

HRMS

Method B: m/z $[M+H]^+$ calcd. for $[C_6H_7N_2O_2S]$: 171.0228 found: 171.0224.

NMR Spectroscopy

^{1}H NMR (500 MHz, DMSO-d_6) δ 7.02 (dd, *J* = 7.9, 4.5 Hz, 1H), 3.99 (dd, *J* = 12.9, 7.9 Hz, 1H), 3.65 (dd, *J* = 12.9, 4.5 Hz, 1H), 2.08 (s, 3H).

^{13}C{^{1}H} NMR (75 MHz, DMSO-d_6) δ 168.7, 147.7, 111.8, 96.8, 38.4, 20.6

IR Spectroscopy

IR vmax (cm^{-1}): 3015 (w, C-H), 2952 (w, C-H), 2242 (w, C≡N), 1752 (s, C=O), 1572 (m, C=N), 1427 (w, C-H), 1372(m, C-C), 1343 (w, C-H), 1211 (s, C-H), 1142 (m, C-O), 1105 (s, C-N), 1045 (m), 1014 (m)

^{1}H NMR Spectrum

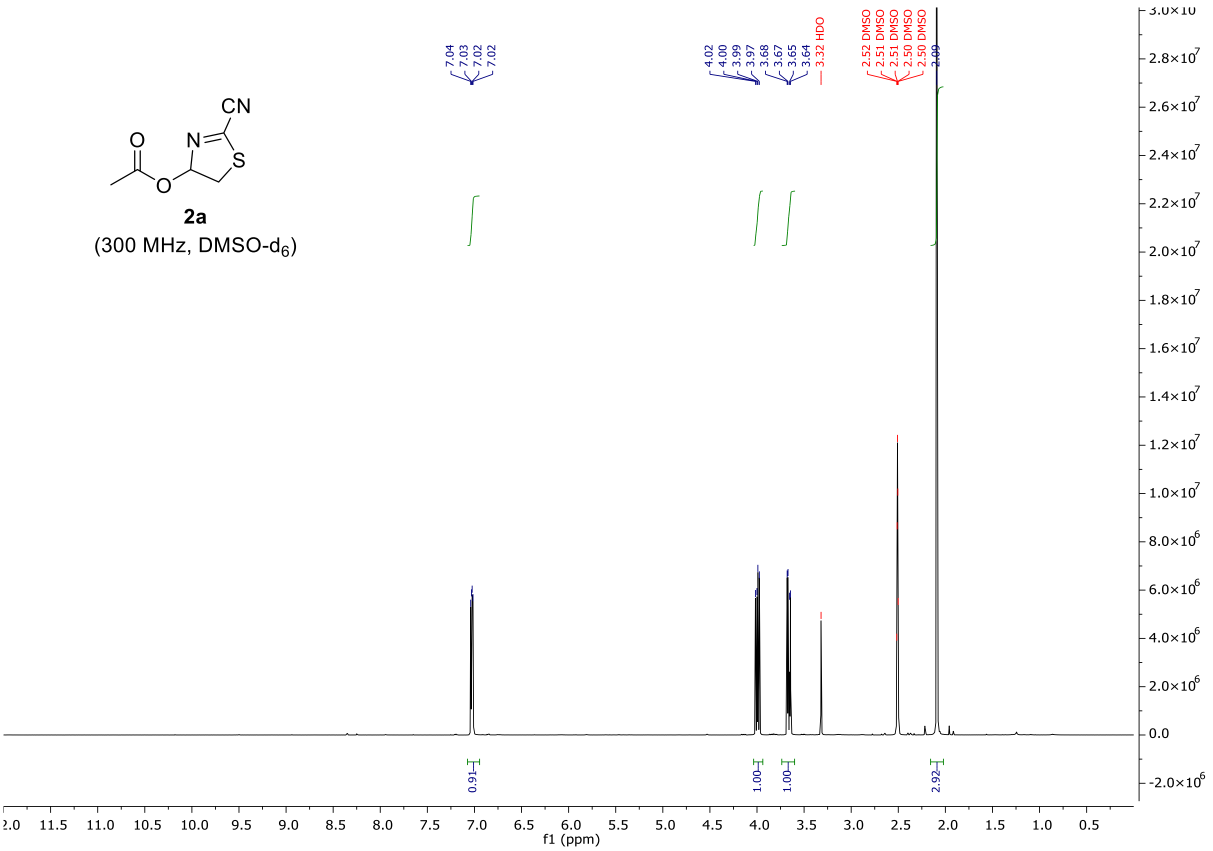

^{13}C{^{1}H} NMR Spectrum

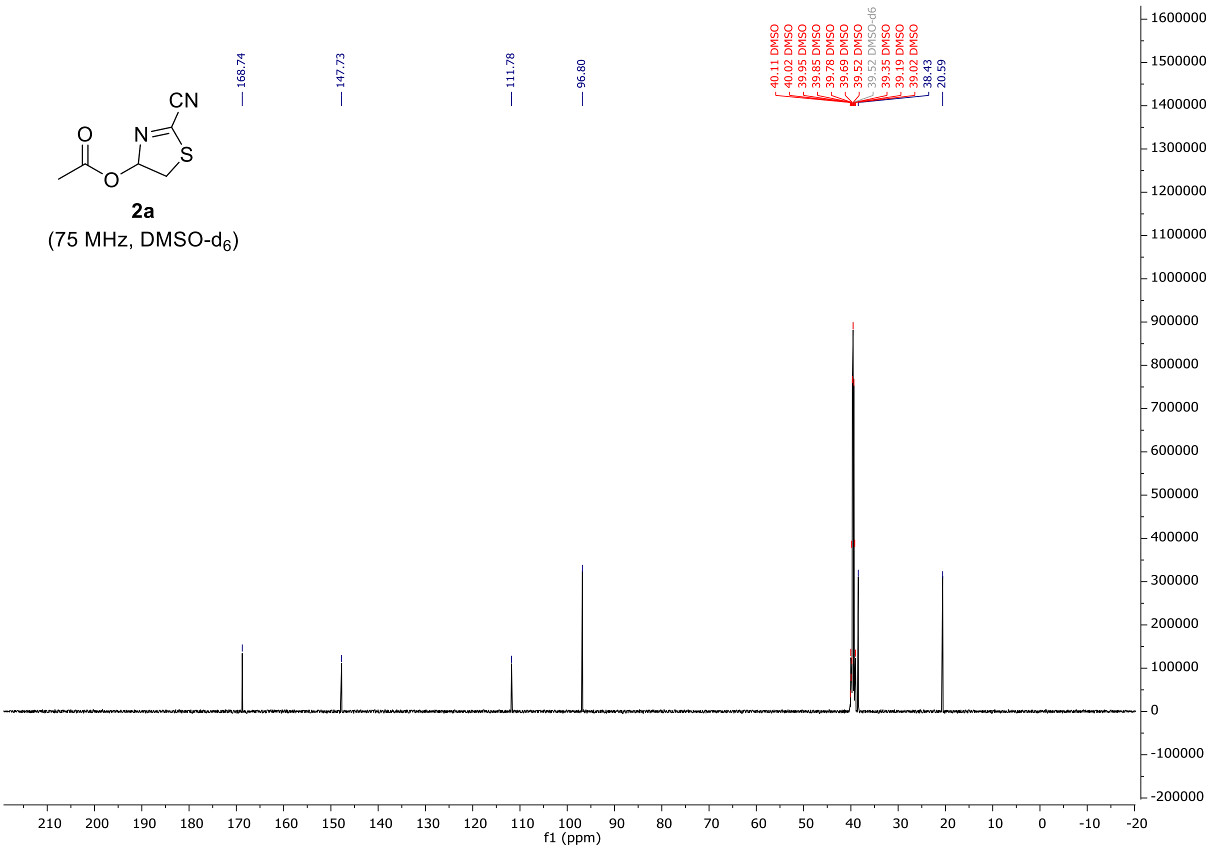

4.3 2-Cyano-4,5-dihydrothiazol-5-yl Benzoate (2b)

HPLC Analysis

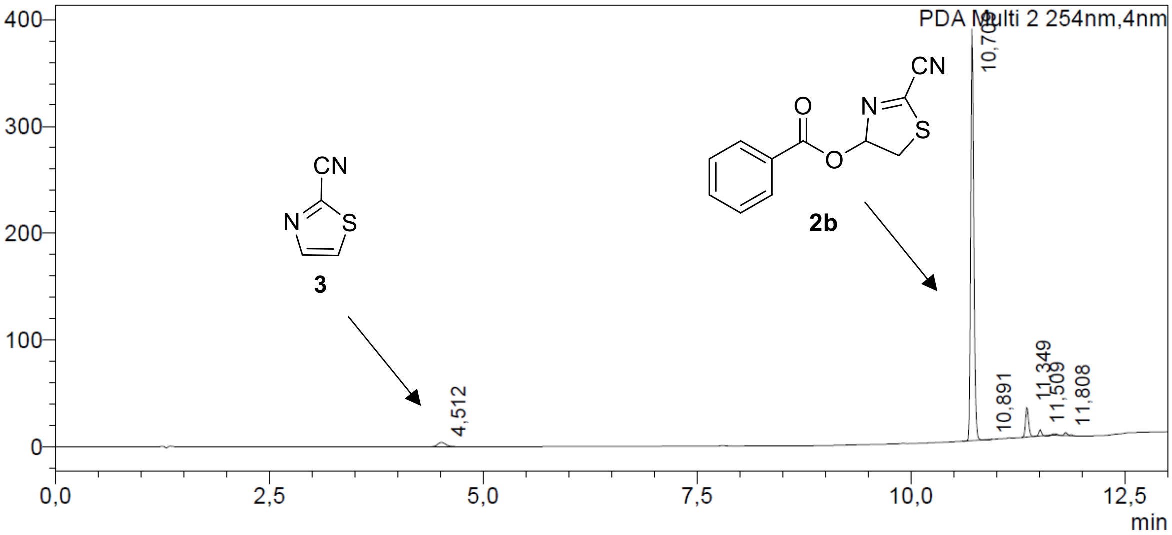

HPLC trace of title compound after column chromatography.

Retention Time: 10.709 min

HPLC Purity: 88.6 %

HRMS

Method A: m/z [M+Na] calcd. for [$C_{11}H_8N_2O_2SNa$] 255.0199 found 255.0201.

Melting Point

93.9 – 96.1 °C

NMR Spectroscopy

^{1}H NMR (300 MHz, DMSO-d_6) δ 7.99 (d, *J* = 7.2 Hz, 2H), 7.71 (t, *J* = 7.4 Hz, 1H), 7.55 (t, *J* = 7.7 Hz, 2H), 7.29 (dd, *J* = 7.9, 4.7 Hz, 1H), 4.11 (dd, *J* = 12.9, 7.9 Hz, 1H), 3.88 (dd, *J* = 12.9, 4.6 Hz, 1H).

^{13}C{^{1}H} NMR (75 MHz, DMSO-d_6) δ 164.0, 148.1, 134.0, 129.6, 128.9, 128.6, 111.9, 97.7, 38.5.

IR Spectrsocopy

IR vmax (cm^{-1}): 3018 (w, C-H), 2967 (w, C-H), 2250 (w, C≡N), 1719 (s, C=O), 1599 (w, C=C), 1586(w, C=C), 1552 (m, C=N), 1491 (w), 1452 (w), 1422 (w), 1341 (w), 1314 (w), 1259 (m), 1239 (m), 1177 (m), 1089 (m), 1066 (m), 1040 (m), 1026 (m)

^{1}H NMR Spectrum

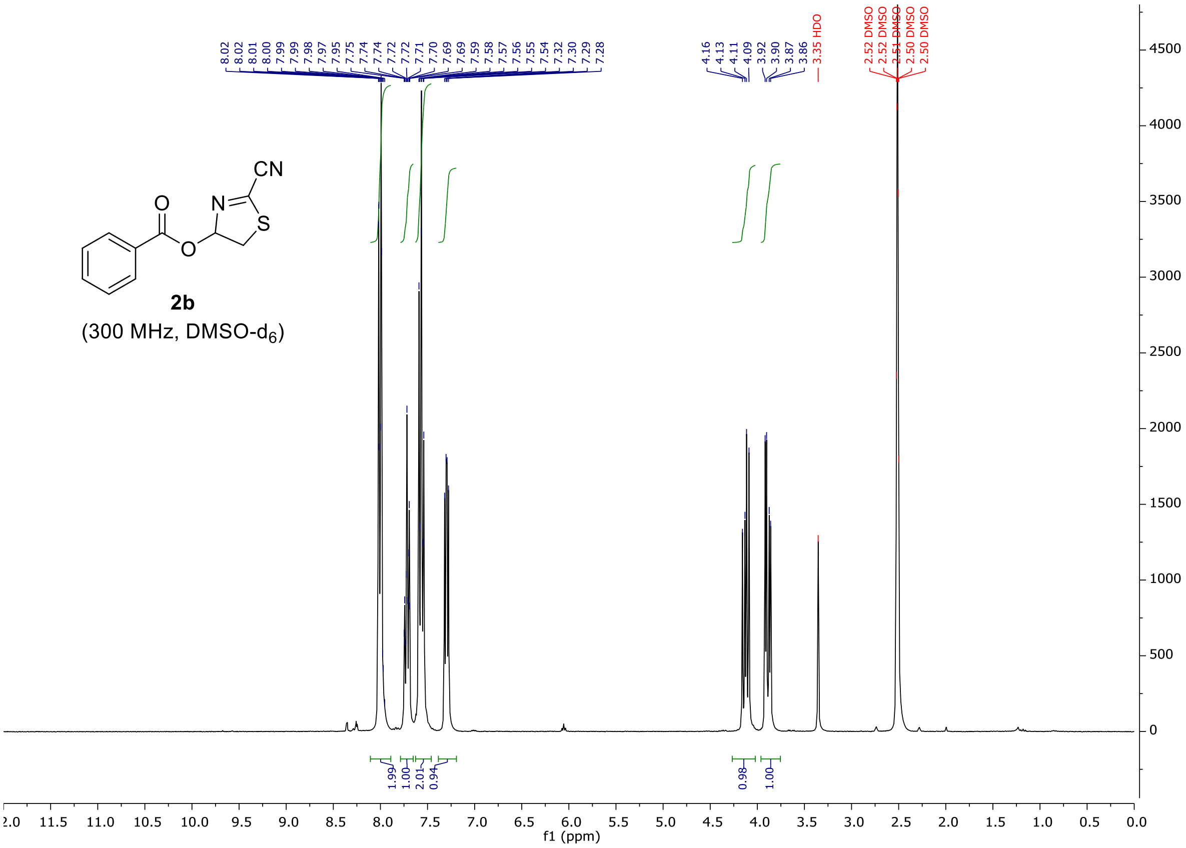

^{13}C{^{1}H} NMR Spectrum

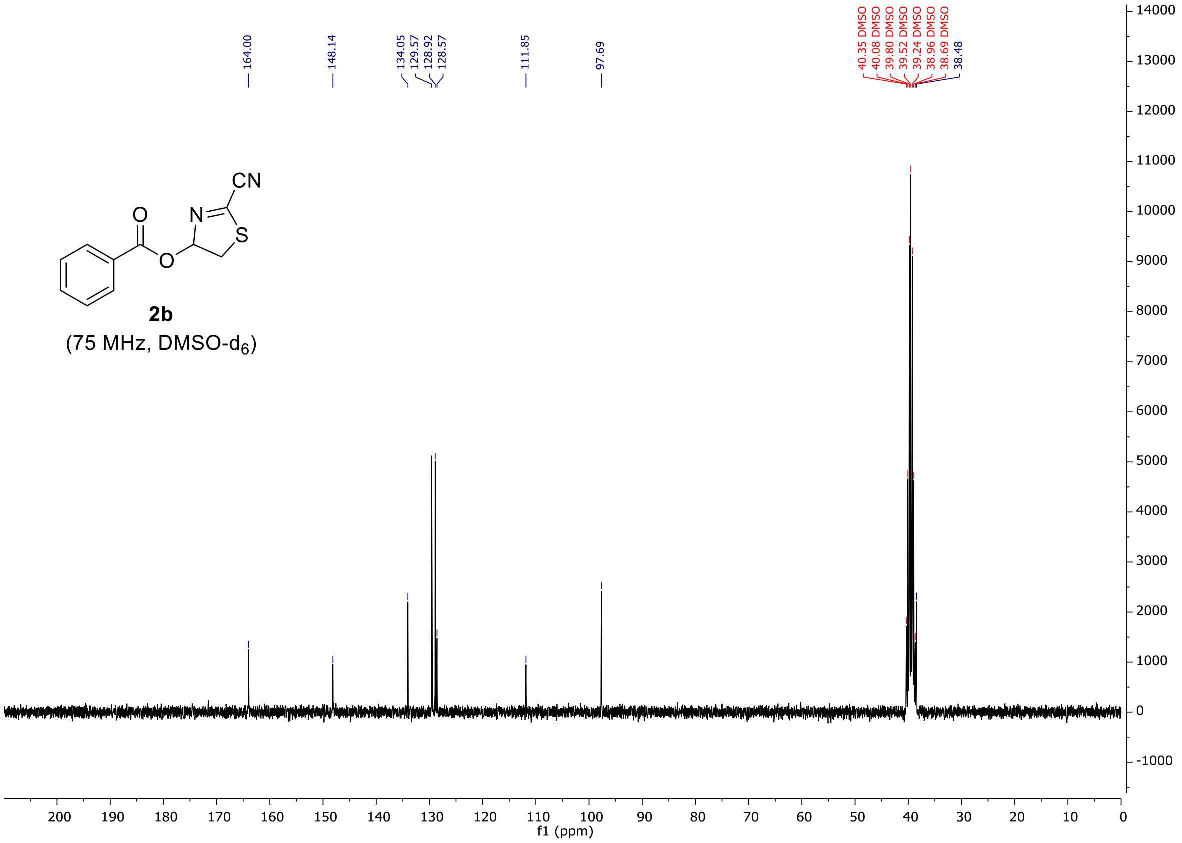

4.4 Synthesis of 2-Cyanothiazole (3)

HPLC Analysis

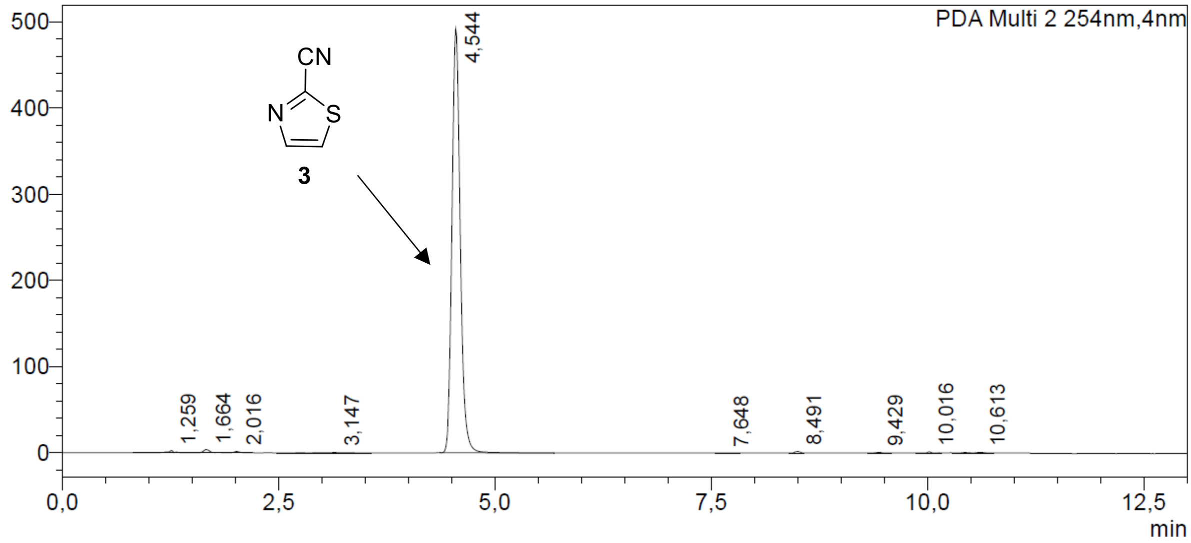

Retention Time: 4.544 min

HPLC Purity: 97.8%

Melting Point

29.6 – 31.1 °C

NMR Spectroscopy

^{1}H NMR (300 MHz, DMSO-d_6) δ 8.34 (d, *J* = 3.1 Hz, 1H), 8.24 (d, *J* = 3.1 Hz, 1H).

^{13}C NMR (75 MHz, DMSO-d_6) δ 145.4, 135.8, 128.5, 113.4.

IR Spectroscopy

IR vmax (cm^{-1}): 3115 (m, C-H), 3092 (w, C-H), 2232 (m, C≡N), 1527(w, C=C), 1467 (m, C=N), 1370 (m), 1317 (w, C-N), 1193 (w), 1126 (m), 1061 (m), 1010 (w)

IR and NMR data are in agreement with literature reports.[4]

^{1}H NMR Spectrum

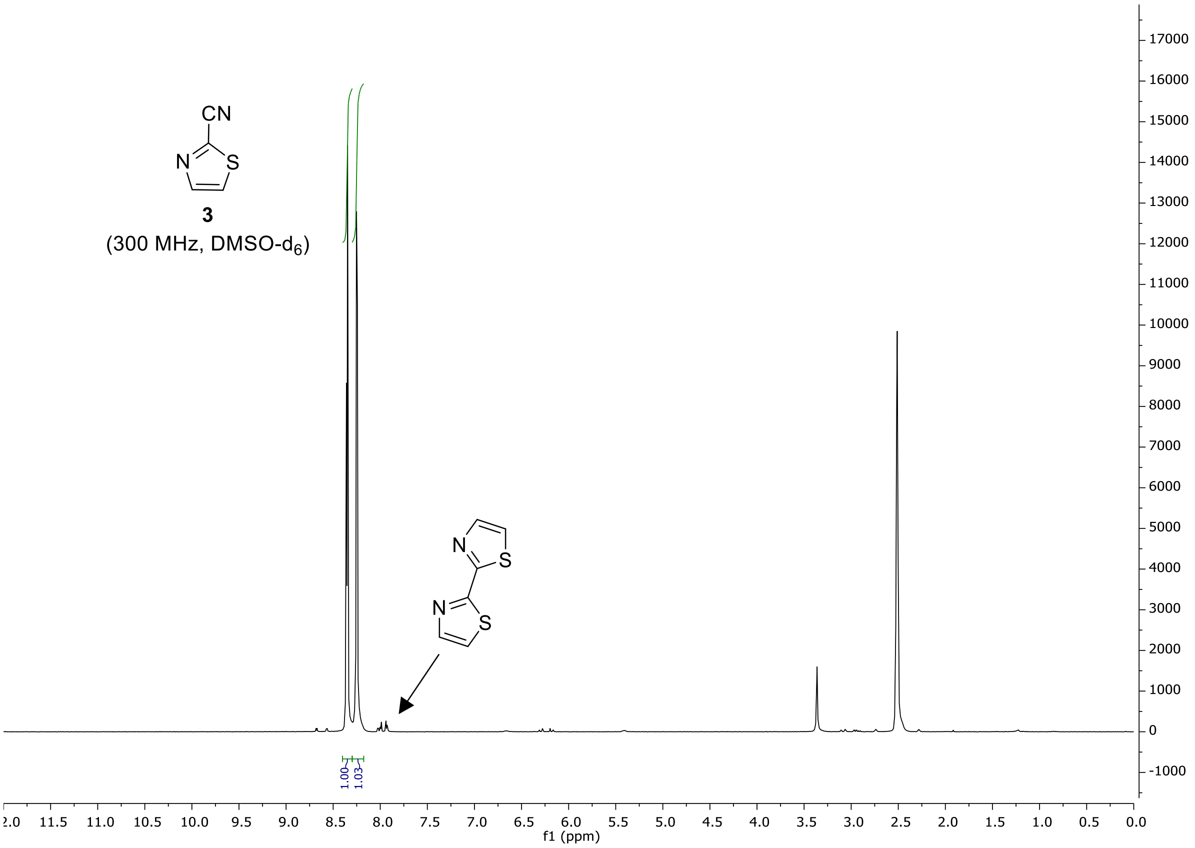

* Contains ~1 mol% of pseudo-dimer.

^{13}C{^{1}H} NMR Spectrum

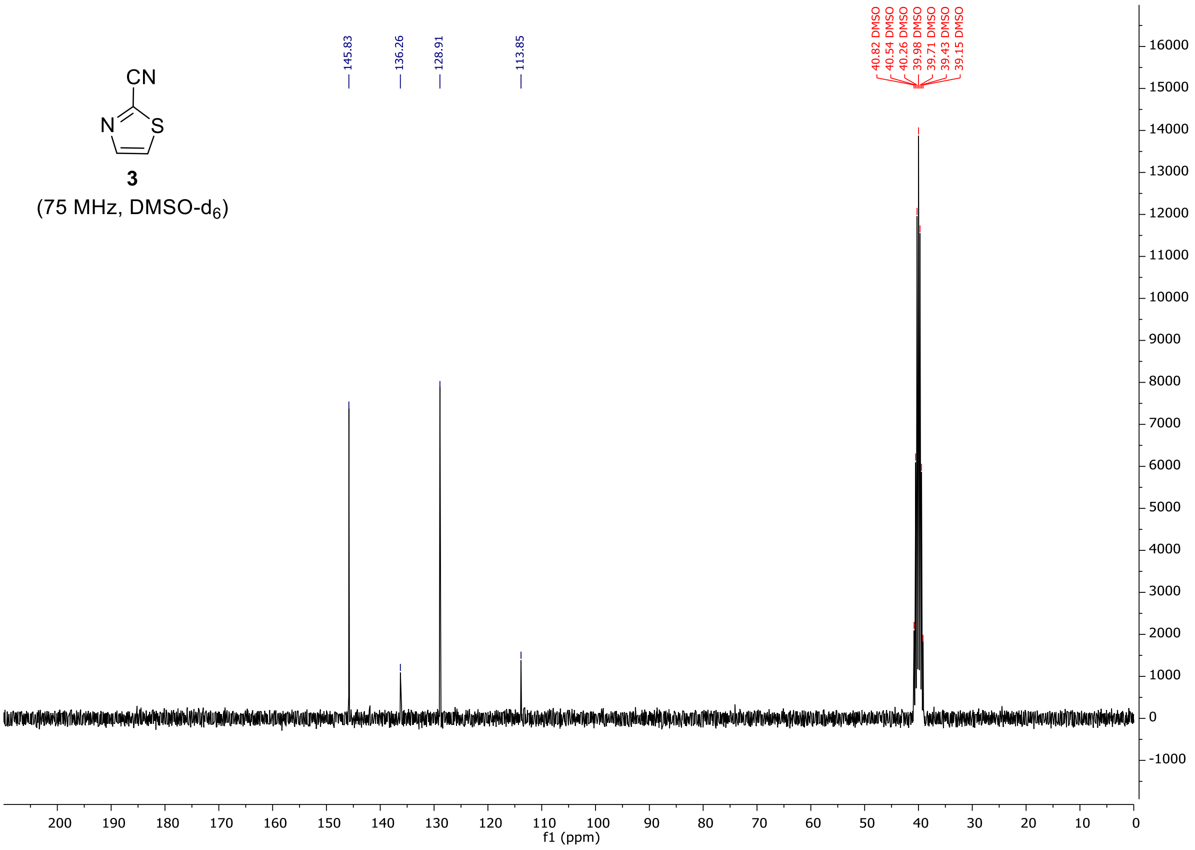

4.5 Thiazole-2-carboximidamide hydrochloride (5)

HPLC Analysis

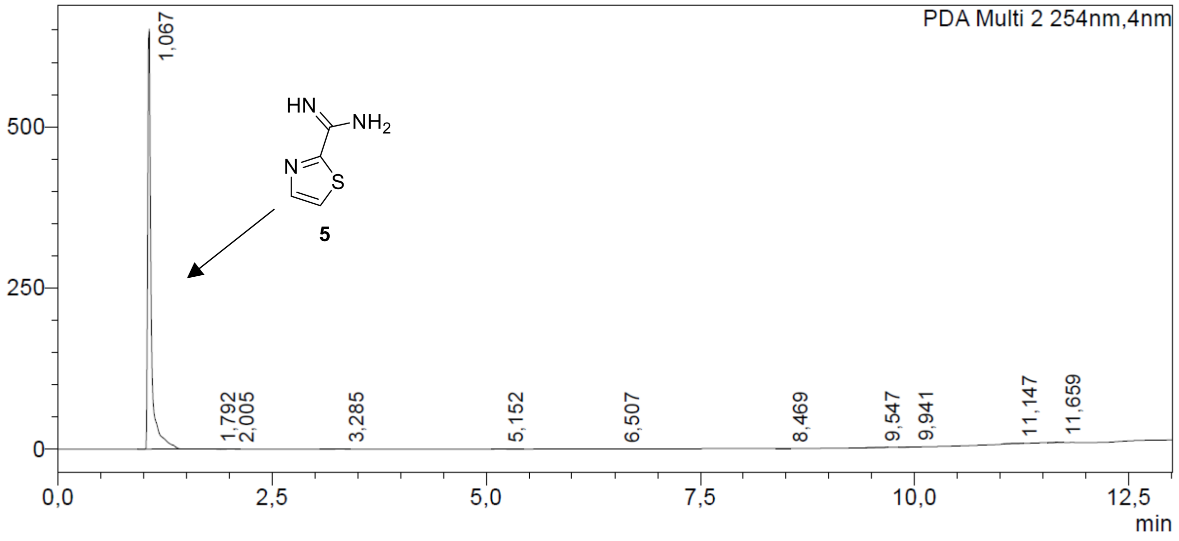

HPLC trace of the crystalline product dissolved in MeOH.

Retention Time: 1.067 min

HPLC Purity: 99.3%

Melting Point

191-193 °C (decomposition)

NMR Spectroscopy

^{1}H NMR (300 MHz, DMSO-d$_6$) δ 9.62 (bs, 3H), 8.38 (d, *J* = 3.0 Hz, 1H), 8.24 (d, *J* = 3.0 Hz, 1H), 7.86 (bs, 1H).

^{13}C{^{1}H} NMR (75 MHz, DMSO-d$_6$) δ 157.0, 154.0, 144.8, 128.8.

IR Spectroscopy

IR vmax (cm^{-1}): 3342 (w, N-H), 3102 (w, C-H), 2957 (br, N-H), 1681 (m, C=N), 1641 (w, C=N), 1548 (w, C=C), 1525 (w), 1470(w), 1398 (w), 1366 (w), 1325(w), 1161 (w), 1138(w), 1100(w), 1053(w).

NMR data in agreement with literature.[5]

^{1}H NMR Spectrum

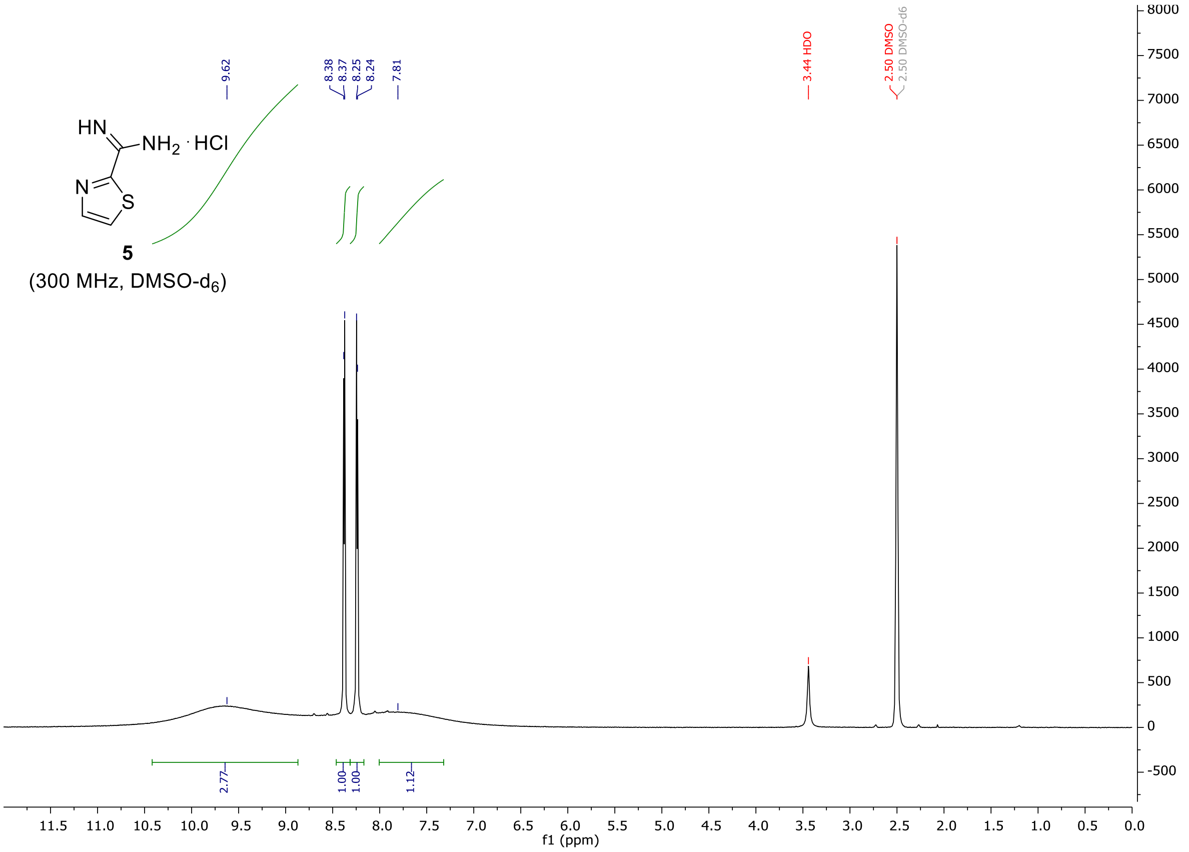

^{13}C{^{1}H} NMR Spectrum

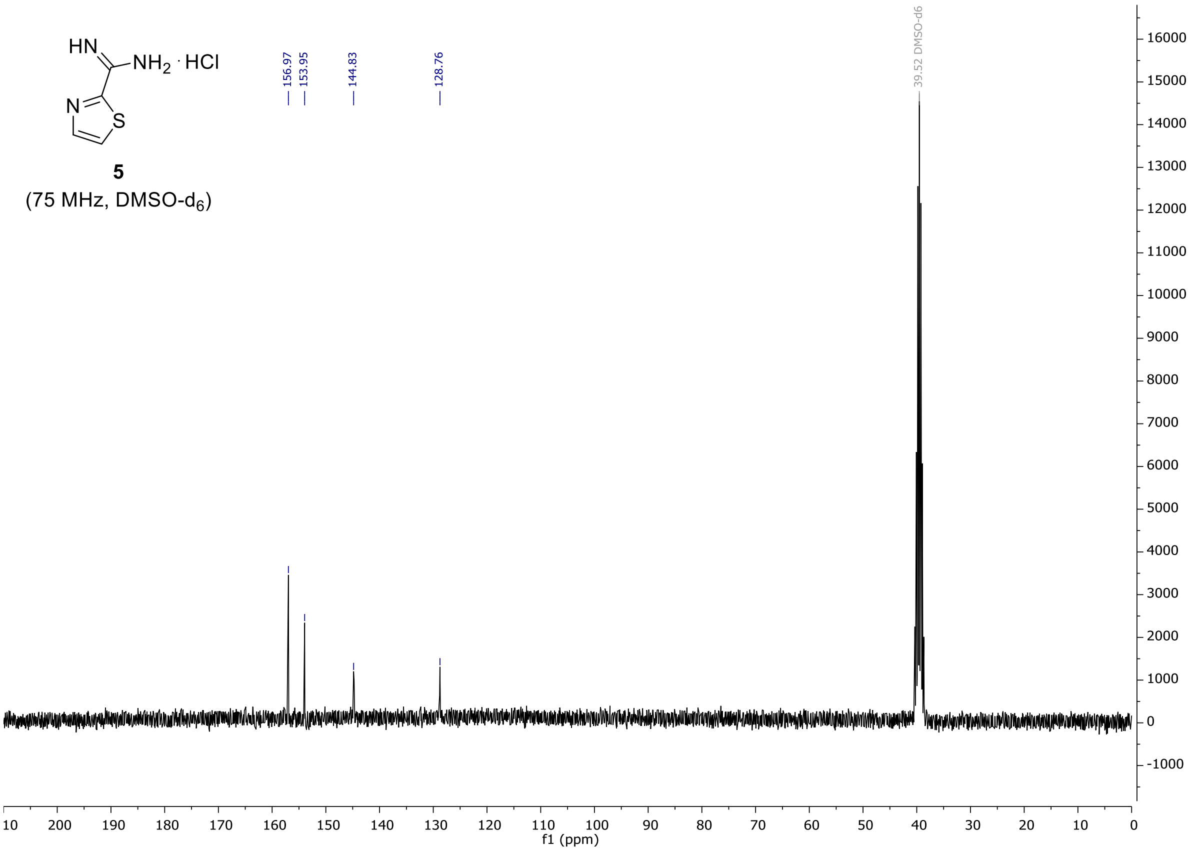

5. References

(1) Wang, Y. L.; Lee, H. D.; Beach, M. W.; Margerum, D. W. Kinetics of Base Hydrolysis of Cyanogen and 1-Cyanoformamide. *Inorg. Chem.* **1987**, *26*, 2444–2449.

(2) Köckinger, M.; Hone, C. A.; Kappe, C. O. HCN on Tap: On-Demand Continuous Production of Anhydrous HCN for Organic Synthesis. *Org. Lett.* **2019**, *21*, 5326–5330.

(3) a) McNerney, J. M.; Schrenk, H. H.; The acute Toxicity of Cyanogen *Am. Ind. Hyg. Assoc. J.* **1960**, *21:2*, 121-124. b) Singh, J.; Kaushik, R. D.; Chawla, M. Hazardous gases Risk Assessment on the Environment and Human Health, 1st edition, Academic Press, **2021**, pp. 320.

(4) Sayama, S. Synthesis of Nitriles from Aldehydes with Trimethylphenylammonium Tribromide and Ammonium Acetate. *Heterocycles* **2016**, *92*, 1796–1802.

(5) Li, X.; Zhou, K.; He, H.; Zhou, Q.; Sun, Y.; Hou, L.; Shen, L.; Wang, X.; Zhou, Y.; Gong, Z.; et al. Design, Synthesis, and Evaluation of Tetrahydropyrrolo[1,2- c]Pyrimidines as Capsid Assembly Inhibitors for HBV Treatment. *ACS Med. Chem. Lett.* **2017**, *8*, 969–974.
